# Low-resolution description of the conformational space for intrinsically disordered proteins

**DOI:** 10.1038/s41598-022-21648-9

**Published:** 2022-11-09

**Authors:** Daniel Förster, Jérôme Idier, Leo Liberti, Antonio Mucherino, Jung-Hsin Lin, Thérèse E. Malliavin

**Affiliations:** 1 UMR7374 Interfaces, Confinement, Matériaux et Nanostructures, Université d’Orléans, Orléans, France; 2 UMR6004 Laboratoire des Sciences du Numérique de Nantes, Nantes, France; 3 LIX UMR 7161 CNRS École Polytechnique, Institut Polytechnique de Paris, 91128 Palaiseau, France; 4 IRISA, University of Rennes 1, Rennes, France; 5 Biomedical Translation Research Center, Academia Sinica, Taipei, Taiwan; 6 Institut Pasteur, Université Paris Cité, CNRS UMR3528, Unité de Bioinformatique Structurale, F-75015 Paris, France; 7 Université de Lorraine, CNRS UMR7019, LPCT, F-54000 Nancy, France

**Keywords:** Solution-state NMR, Computational models, Statistical methods, Protein structure predictions

## Abstract

Intrinsically disordered proteins (IDP) are at the center of numerous biological processes, and attract consequently extreme interest in structural biology. Numerous approaches have been developed for generating sets of IDP conformations verifying a given set of experimental measurements. We propose here to perform a systematic enumeration of protein conformations, carried out using the TAiBP approach based on distance geometry. This enumeration was performed on two proteins, Sic1 and pSic1, corresponding to unphosphorylated and phosphorylated states of an IDP. The relative populations of the obtained conformations were then obtained by fitting SAXS curves as well as Ramachandran probability maps, the original finite mixture approach RamaMix being developed for this second task. The similarity between profiles of local gyration radii provides to a certain extent a converged view of the Sic1 and pSic1 conformational space. Profiles and populations are thus proposed for describing IDP conformations. Different variations of the resulting gyration radius between phosphorylated and unphosphorylated states are observed, depending on the set of enumerated conformations as well as on the methods used for obtaining the populations.

Intrinsically disordered proteins (IDP) are at the center of the attention in the structural biology of proteins. Indeed, disordered residues are expected to constitute 35 to 50% of the human proteome and, depending on the organism type, the overall percentage of amino acids predicted to be disordered ranges from about 12% up to 50%^1^. In addition, the conformational plasticity of the disordered regions of proteins allows them to interact with numerous partners in the cell, as for example for the three intrinsically disordered domains of the tumor protein P53^2^. This moonlighting^3^ behavior explains the strong impact of IDPs in cellular signaling, regulation, and control, and the differences observed in their interactomes with respect to globular proteins^4^.

Intrinsically disordered proteins represent a challenge for structural biology for several reasons. In solution, the nuclear Overhauser effects measuring distance between hydrogens are usually not available. Moreover, crystallization processes are hampered by the conformational disorder, or the variability of conformations in the crystal or in the electron cryogenic maps makes impossible the observation of electronic density for disordered regions. Numerous approaches have been proposed^5–8^ for the calculation of protein conformations, based on molecular dynamics or Monte Carlo simulations for generating molecular conformations.

We propose here to explore a new approach for the exploration of the conformational space of IDPs, based on a systematic enumeration of conformations in the frame of the distance geometry problem. We build on our previous work introducing TAiBP as a new tool to investigate structural ensembles of IDPs in a systematic way, by predicting populations and consequently selecting pools of representative conformations. This approach, initiated as the interval Branch-and-Prune (iBP) algorithm by Mucherino and coworkers^9^, was adapted to the protein molecular modeling as threading-augmented interval Branch-and-Prune (TAiBP)^10,11^. Based on distance geometry, TAiBP explores the entire conformational space compatible with NMR chemical shifts, retaining conformations that are most different from one another, and thus yielding a diverse set of conformations to be analyzed further. This is in contrast to Monte Carlo methods which are informed by force fields and explore the part of the configurational space that is thermodynamically relevant in more detail. TAiBP was recently shown^12^ to allow the analysis of the conformational space of a tandem domain of protein whirlin, in which a disordered linker induces a large orientation variability of two PDZ domains^13^. The application of TAiBP to the tandem domain was made possible by the analysis of unprocessed output of the neural network TALOS-N^14^, the Ramachandran likelihood maps. Indeed, drawing boxes on the most probable regions of these maps allowed the determination of intervals on backbone angles, which serve as inputs for the TAiBP algorithm. It should be noticed that the approach MERA has been developed^15^ for the prediction of the $\phi$, $\psi$ distributions for IDPs.

In the present work, we apply TAiBP to a well-know example of IDP^16,17^. The obtained IDP conformations will be filtered and their relative populations determined by BioEn^18^ using SAXS data. In parallel, we propose an original method, RamaMix, to select the main conformations, as well as their populations, according the Ramachandran likelihood maps predicted by TALOS-N^14^. The principle of RamaMix is to fit a bivariate, periodic, finite mixture model to the output of TALOS-N. The *N* terminal fragment of the intrinsically disordered protein Sic1, as well as its phosphorylated form pSic1, each one spanning 90 residues, will be studied.

Sic1 prevents premature S-phase entry in the budding yeast *Saccharomyces cerevisiae* by inhibiting the complex Cdk1-Clb. At the START point in the yeast cell cycle, Sic1 is phosphorylated on three Threonines (residues 7, 35, and 47) and three Serines (residues 71, 78, and 82) in order to be degraded by the proteosome. Sic1 as well as pSic1 were shown^16,19^ to contain significant amount of transient secondary structures.

The comparison of repeated runs of TAiBP on Sic1 and pSic1 reveals a good reproducibility of global conformational shape. Qualitatively similar but quantitatively different populations are obtained either by fitting distinct SAXS curves or Ramachandran maps. The sets of individual conformations selected from the fitting of various data are partially distinct, but better convergence is observed for the profiles of local gyration radius. These profiles could be proposed as a low resolution description of the IDP conformational space. Depending on the way the TAiBP conformations are generated, and on the processing method to obtain the populations, different patterns of variations are observed for the resulting gyration radius of Sic1 and pSic1.

## Results

### Enumeration of protein conformations

The TALOS-N^14^ prediction was obtained using the chemical shifts measured for the nuclei H$\alpha$, HN, $^{15}$N, $^{13}$C$\alpha$, $^{13}$C$\beta$ of Sic1 and pSic1 residues, and was used to determine boxes of $\phi$ and $\psi$ values, giving the limits in which the conformations will be enumerated. Indeed, from the NMR chemical shifts and the protein sequence information, the TALOS-N neural network predicts the likelihood that a given residue *n* has backbone torsion angles that fall in any of the 324 voxels, of 20$^{\circ }$ $\times$ 20$^{\circ }$ each, that make up the Ramachandran map^14^. Following the approach proposed in Ref. ^12^, we define boxes (Figs. S1–S4) using Ramachandran regions displaying largest likelihood for the TALOS-N prediction, and corresponding supposedly to protein conformations populated in solutions.

In order to probe the reliability of the ($\phi$,$\psi$) boxes obtained from the TALOS-N likelihood maps, these boxes were compared to the predictions performed using the approach MERA^15^, which predicts the residue-by-residue Ramachandran map distributions for disordered proteins using short-range NOEs, chemical shifts, J couplings and spectral density derived from the N$^{15}$ relaxation measurement. As only chemical shifts were available for Sic1 and pSic1, the MERA prediction was performed putting all other possible inputs to zero. The MERA Ramachandran map distributions are plotted for all successful predicted residues, along with the input boxes derived from the TALOS-N prediction (Figs. S5 and S6), showing a reasonable agreement between the two methods.

Two replicates of boxes were generated for Sic1 and pSic1, using threshold values of 0.01 and 0.011 on the Ramachandran probability maps as described in Section “Extraction of boxes from Ramachandran likelihood” in the Supplementary Material. Using these sets of input boxes, five TAiBP runs were performed, named Sic1$^1$, Sic1$^2$, pSic1$^1$, pSic1$^2$ and pSic1$^3$. The run pSic1$^3$ was using the input boxes of pSic1$^2$, but differs from the other runs by the procedure for selecting more extended representative conformations after the SOM clustering, as described in the section “Clustering of generated conformations” in the Supplementary Information.

The two replicates of TAiBP calculations introduced in the previous subsection were based on similar numbers of fragments: 14 and 13 for Sic1$^1$ and Sic1$^2$, 17 for pSic1$^1$ and pSic1$^2$ and 18 for pSic1$^3$ (Table S1). The larger number of fragments used for pSic1 arises from the regions of residues 5-9, 33-37, 45-49, 69-73, 76-84 for which TALOS-N was unable to give a prediction due to the phosphorylated residues and for which generic boxes (Table S2) were used. These boxes being formed of three components, they increase the combinatorics of the enumeration and shorter fragments have to be used, requiring a larger number of fragments to span the protein sequence.

**Figure 1:**
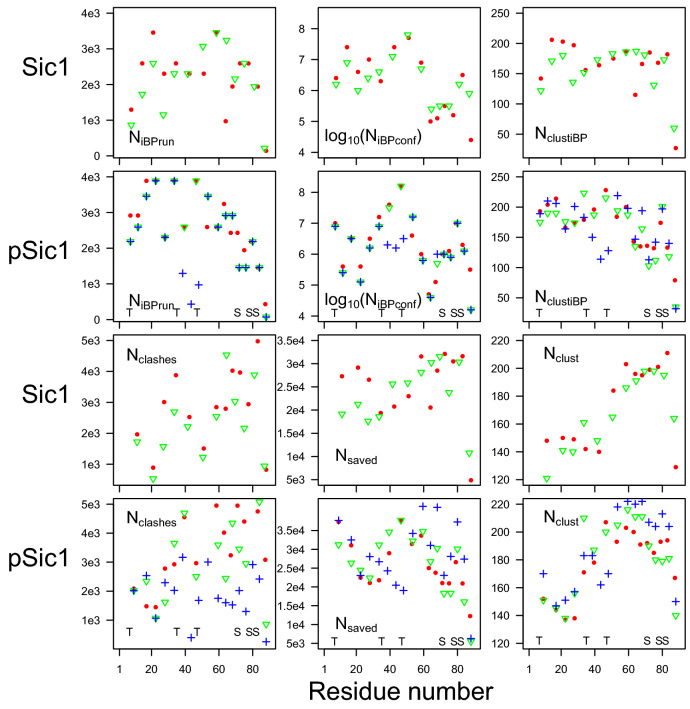
Parameters of the iBP and assembly steps of the TAiBP procedure. The signs red and green correspond respectively to the duplicated runs in which thresholds of 0.01 and 0.011 have been applied on the probability Ramachandran map. The blue crosses correspond to the run pSic1$^3$ producing more extended conformations. The positions of phosphorylated Threonines and Serines are marked with T and S for the runs on pSic1. The parameters are plotted along the number of the residue located at the middle of the fragment (iBP step) or at the middle of the last attached fragment (assembly step).

The boxes used as inputs for the TAiBP runs (Figs. S1–S4) are quite similar. The loop region (positive $\phi$) is slightly more populated for runs pSic1$^1$ and pSic1$^2$. For the iBP and assembly steps forming the TAiBP approach, the runs, marked in colors red, green and blue in Fig. 1, produce parameter values similar in most of the protein sequence.

For the iBP steps, three parameters were compared (Fig. 1, first and second lines) along the residue number located at the middle of each fragment: the number of individual iBP runs ($N_\text {iBPrun}$), the number of saved conformations ($N_\text {iBPconf}$) and the number of obtained conformations after clustering ($N_\text {clustiBP}$). The three analyzed parameters are located in similar ranges for all calculations. Nevertheless, $N_\text {iBPrun}$ displays the largest observed values (3888) around the positions of phosphorylated Threonines in agreement with the larger generic boxes used in these protein regions (Table S2). Such an increase is not observed for phosphorylated Serines due to shorter fragments used in the region 50-90 (Table S1). A decrease of $N_\text {iBPrun}$ is observed for pSic1$^3$ around the residue 40, due to the shorter peptide fragments used in this region. For every calculation, $N_\text {iBPconf}$ is smaller than $10^9$, which is the input given for the maximum number of solutions: all individual iBP trees have thus been completely parsed. The $N_\text {iBPconf}$ profiles display smaller values, mostly in the range 10$^6$-10$^7$, for all calculations in the region of residues 60-90. Contrarily to $N_\text {iBPconf}$, the numbers of clustered conformations ($N_\text {clustiBP}$) display relatively flat profiles for Sic1, but a decrease in the number of conformations of pSic1 in the region of residues 60-90. This larger reduction of conformations due to the clustering is the sign that the conformations generated by iBP in the region 60-90 are more diverse in Sic1 than in pSic1. In all calculations, the C terminal fragments which are smaller than the others (Table S1) display smaller $N_\text {iBP}$, $N_\text {iBPconf}$ and $N_\text {clustiBP}$. The results obtained for the run pSic1$^3$ (blue crosses) are quite similar to those of the run pSic$^2$, which is not surprising as the fragment definition are the same, except around residues 40-60 (Table S1).

Three parameters are plotted (Fig. 1, third and fourth lines) along the assembled fragments: the number of conformations rejected due to C$\alpha$ atoms closer than 1Å ($N_\text {clashes}$), the number of saved conformations ($N_\text {saved}$) and the number of clustered conformations ($N_\text {clust}$). Looking at the relative ranges of values of $N_\text {clashes}$ and $N_\text {saved}$, between 10% and 15% of the assembled fragments are rejected due to the steric clashes. A smaller percentage of rejection is observed for pSic1$^3$ (blue crosses): the more extended conformations display a tendency to produce less clashes. The profiles of $N_\text {clust}$ are different for Sic1 and pSic1, as the number of clustered conformations increases up to the last fragment, whereas this number already starts to decrease in the region of residues 60-90 in pSic1. This effect can be put in parallel with the decrease of $N_\text {clustiBP}$ in the same region during the iBP step. The last fragments of proteins have strong decreasing effects on $N_\text {clust}$ due to their smaller size (Table S1), which probably induces less variability in the generated conformations. The number of clustered and saved conformations for pSic1$^3$ is often larger than in other runs, which may be a consequence of the smaller numbers of clashes.

After the distance geometry calculations, a refinement by molecular dynamics (MD), described in Section “Molecular dynamics refinement in implicit solvent” of Supplementary Material, was applied to the generated conformations. The protein conformations do not vary much during MD trajectories. Indeed, the cumulative sums of differences between initial and final values of backbone angles produce values in the range 4.2 to $4.9^{\circ }$ for $\phi$ and 0.04 to $2.4^{\circ }$ for $\psi$. Similarly, the average coordinate RMSD between the initial and final frames of the refinement trajectories are 0.6 Å for the four runs Sic1$^1$, Sic1$^2$, pSic1$^1$ and pSic1$^2$. The drift is slightly larger for pSic1$^3$, with backbone angle values in the ranges $-24$ to $6^{\circ }$ for $\phi$ and $-40$ to $-1^{\circ }$ for $\psi$, and an average coordinate RMSD of 0.7 Å. The conformations displaying potential energy smaller than $-50$ kcal/mol for the runs Sic1$^1$ and Sic1$^2$ and smaller than $-600$ kcal/mol for the runs pSic1$^1$, pSic1$^2$ and pSic1$^3$, were selected for further analyses. This selection produces sets of 98 (Sic1$^1$), 133 (Sic1$^2$), 161 (pSic1$^1$), 121 (pSic1$^2$) and 148 (pSic1$^3$) conformations.

### Comparison of the conformations between duplicate TAiBP runs

**Figure 2:**
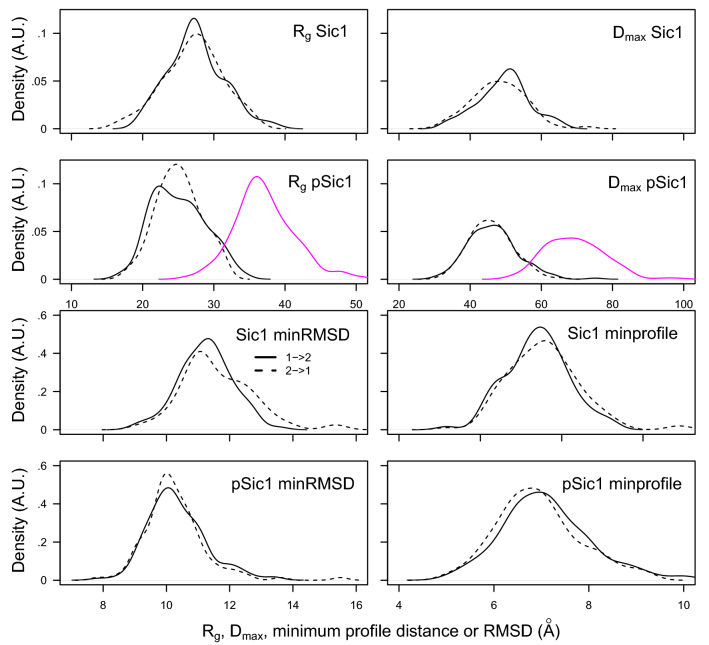
Four panels on top: Distribution of the gyration radii R$_g$ and of maximal diameters D$_\text {max}$ values in the two sets of TAiBP obtained during the first runs Sic1$^1$ and pSic1$^1$ (solid line) and the second runs Sic1$^2$ and pSic1$^2$ (dashed line) runs. The R$_g$ and D$_\text {max}$ distribution obtained for the run pSic1$^3$ are plotted in magenta. Four panels on bottom: Distribution of the minimum RMSD values (Å) and of the minimum distances (Å) between profiles for the duplicate runs performed for Sic1$^1$ and Sic$^2$ and for pSic1$^1$ and pSic1$^2$. full line: first run with respect to the second one, dashed line: second run with respect to the first one.

The distributions of gyration radii R$_g$ and maximal diameters D$_\text {max}$ (Fig. 2 two top rows) are quite similar for the duplicate runs on Sic1 and pSic1. The global envelope of generated conformation is thus reproducible between the replicated TAiBP runs. The distributions of gyration radii R$_g$ and maximal diameters D$_\text {max}$ have been plotted in magenta for the run pSic$^3$ to display the larger extension of the obtained conformations.

The individual conformations generated for the duplicated runs of Sic1 and pSic1 were then compared by calculating the two-by-two coordinate root-mean-square deviation (RMSD, Å). The distributions of the minimum RMSD values (Fig. 2 two bottom left panels) observed for each conformation of one run to the conformations of the other run are quite reproducible whatever is the performed comparison. They display sets of values in the ranges of 8-16 Å for both proteins, with a maximum around 11 Å for Sic1 and around 10 Å for pSic1. This range 8-16 Å means that the individual conformations of a given run are not reproducible in the replicated run. This excludes a high resolution determination of representative conformations, which is not surprising due to the enormous size of the conformational space to explore and the heavy clustering procedure used along the TAiBP approach.

By analogy to the cross-sectional gyration radius, we propose here the profiles of local gyration radii to describe the local variation in the shape of conformations. These profiles $P_q$ of local gyration radii are calculated along residue number *n* for each conformation *q* in the following way:

1 $$\begin{aligned} P_q(n) = \sqrt{\frac{1}{N_n} \sum _{i=n-N_\text {win}}^{n+N_\text {win}} ({\textbf {X}}_{i}-{\textbf {X}}^\text {ave}_{n})^2} \end{aligned}$$

where ${\textbf {X}}_{i}$ represents the vector of atomic coordinates for the backbone atoms of residue *i* in the range $n-N_\text {win}$, $n+N_\text {win}$, and $N_\text {win}=5$ is the residue window around *n* on which a local gyration radii is calculated, $N_n$ being the number of backbone atoms located in this window. ${\textbf {X}}^\text {ave}_{n}$ is the coordinate vector of the centroid of the atomic coordinates of the backbone atoms of residues in the range $n-N_\text {win}$, $n+N_\text {win}$.

The profiles $P_q$ of local gyration radii were compared two-by-two between conformations using Euclidean distance. The shapes of distributions for minimal distances between $P_q$ (Fig. 2 bottom right panels) are similar to those observed for minimal RMSD values (Fig. 2 two bottom left panels), but are drifted toward ranges of 4-11 Å. The comparison between local gyration profiles shows that one half of the obtained conformations displays a distance between profiles located between 1/6 and 1/3 of the average gyration radius. The profile distance smaller than the average gyration radius is the sign of a reduced variation of the profiles $P_q$ with respect to the coordinate RMSD. The $P_q$ profiles, inspired by the cross-sectional gyration radius, seems thus to capture a better convergence between the duplicate runs than the coordinate RMSD. In the following, the conformations selected by the fitting of SAXS curves and Ramachandran maps will be compared through their $P_q$ profiles.

Quite similar global shape of conformations are populated in the duplicated TAiBP runs. The profiles $P_q$ of local gyration radii display also some similarity. But, the comparison of atomic coordinates reveals a large variability of the individual conformations selected by the TAiBP approach, which is not surprising due to the enormous considered conformational space.

### Validation of the finite mixture model on synthetic data

Once a set of conformations have been selected using TAiBP, one needs to detect the conformations significantly populated and to evaluate their relative populations. Indeed, the systematic enumeration along all possible combination of the $\phi$/$\psi$ boxes induces the generation of conformations spanning a space possibly larger than the conformations effectively populated. The populations were determined, from one side, using BioEn^18^ on SAXS data, and on the other side, using on the Ramachandran maps, a finite mixture model, RamaMix, specially developed for this purpose. We first present in this section a validation of RamaMix on synthetic data. The details of RamaMix computation are presented in the section “Determination of the populations from Ramachandran maps” in Methods and in the Supplementary Material.

**Figure 3:**
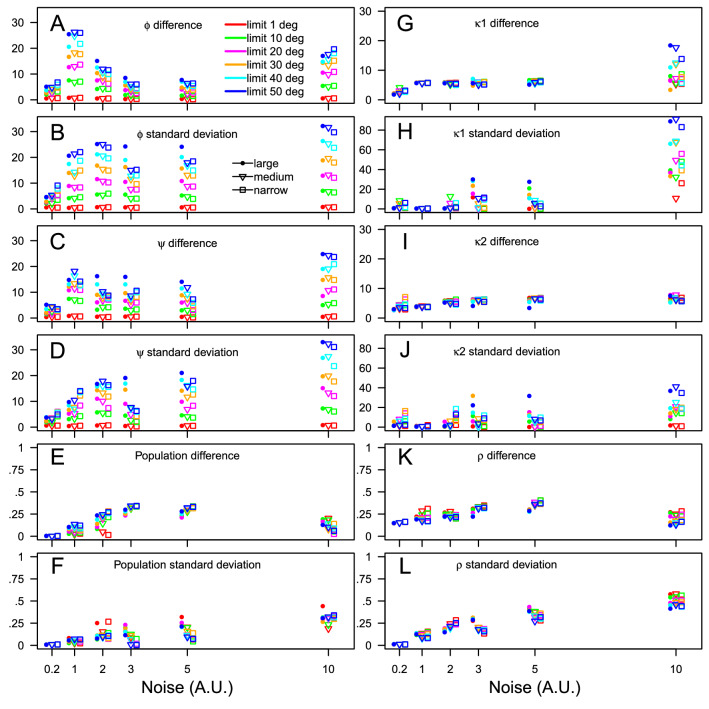
Efficiency of RamaMix for determining the $\phi _0$, $\psi _0$ positions (**A**–**D**: Eq. 7), the von Mises shape parameters $\kappa$1, $\kappa$2 and $\rho$ (**G**–**L**: Eq. 8), and the populations $\gamma _{q}$ (**E**–**F**: Eq. 2) using synthetic data and various noise levels described in Fig. S7. The results obtained for large, medium and narrow scattered synthetic Ramachandran maps are drawn as bullets, triangles and squares.

A pseudo Ramachandran map has been generated by randomly choosing up 15 couples of $\phi$, $\psi$ values located in most populated regions of the Ramachandran map (Fig. S7). Several sets of more or less scattered values, represented by different colors, have been generated, to investigate the effect of conformational superimposition on the population determination. Corresponding populations were also chosen randomly (see caption of Fig. S7). Noise levels of 0.2, 1, 2, 3, 5 and 10 were added to the histogram obtained from the pseudo Ramachandran map, the maximum value of the histogram being around 15. The starting points for each RamaMix run was the $\phi _0$, $\psi _0$ values from the synthetic Ramachandran plot, and random population values. During each RamaMix run, several upper limits were imposed to the drift of the backbone angles during the optimization, with values of: 1$^{\circ }$, 10$^{\circ }$, 20$^{\circ }$, 30$^{\circ }$, 40$^{\circ }$ and 50$^{\circ }$. For each Ramachandran synthetic map, each noise level and each drifting limit value, one hundred runs are performed producing sets of backbone angles ($\phi _0$ and $\psi _0$) (Eq. 7), von Mises parameters (Eq. 8) ($\kappa$1, $\kappa$2 and $\rho$) and populations $\gamma _{q}$ (Eq. 2). Over the 12600 individual RamaMix runs, only 275 runs were terminated without convergence of the optimization. Averages and standard deviations were calculated from the sets of obtained parameters. The differences between the averaged and the input values, as well as the standard deviations (Fig. 3) are used to evaluate RamaMix.

The differences between average and initial populations (Fig. 3E) as well as the standard deviations of populations are mostly smaller than 30%. Thus, the determination of populations is not much influenced by the level of noise, but the population values are rather qualitative. Interestingly, the standard deviation is of the order of value of the difference.

The efficiency of the determination of backbone angles (Fig. 3A–D) for noise levels of 0.2, 1, 2, 3 and 5, is not much influenced by the scattering of synthetic Ramachandran maps, but rather by the drifting limit imposed on the $\phi$, $\psi$ values. Increasing the allowed drift induces larger differences and standard deviations: this would support not allowing large drift for the calculations. Interestingly, for the large scattered Ramachandran map (bullets in Fig. 3), the effect of a large drift is more pronounced than for other synthetic Ramachandran maps. For most of the cases, the standard deviations display larger values than the difference: allowing a drift induces more error on the precision of the calculation than on the average value of angles.

The parameters describing the von Mises distribution (Fig. 3G–L) display contrasted results: the differences are larger for $\rho$ than for $\kappa$1 and $\kappa$2. For $\kappa$1 and $\kappa$2, the standard deviations are much larger than the differences whereas they are similar for $\rho$. The differences between $\rho$ and $\kappa$1 and $\kappa$2, arise from the definition of these parameters (Eq. 8) in which $\rho$ occupies a different place than $\kappa$1 and $\kappa$2.

### Determination of populations

The TAiBP conformations were fitted to the SAXS curves and Ramachandran probability maps using BioEn^18^ and RamaMix. The following sets of conformations were processed: the conformations obtained from runs Sic1$^1$, Sic1$^2$, pSic1$^1$, pSic1$^2$ and pSic1$^3$, as well as two mixed sets of conformations obtained by pooling the conformations from pSic1$^1$ and pSic1$^3$ and the conformations from pSic1$^2$ and pSic1$^3$. These mixed sets of conformations will be denoted pSic1$^{13}$ and pSic1$^{23}$ and encompass respectively 309 and 269 conformations.

BioEn calculations were performed using each of the three SAXS curves available (Tables 1, 2 and S5). The populations larger than 1% found for a given TAiBP run and the fitting of a given SAXS curve, reveal that the same conformations are repeatedly selected: the conformation numbers selected more than once have been written in bold in the Tables. Most of the conformations selected only once, display populations smaller than 15%. But the populations vary significantly from one analysis to another as for example for the conformation 109 from the run Sic1$^2$ (Table 1B) which display populations of 26.6, 40.9 and 43.8% for the three SAXS curve processing. Normalized $\chi ^2$ values smaller than one are found for each calculation along with null final S$_{KL}$ values, in agreement with the definition of S$_{KL}$ as the Kullback-Leibler divergence^18,20^.

Tables 3 and S6 present the populations obtained by RamaMix from the fitting of the Ramachandran probability maps on the same sets of conformations. The variations of backbone angles $\phi$ and $\psi$ during the RamaMix optimization are smaller than 0.25$^{\circ }$ for $\phi$ and 0.1$^{\circ }$ for $\psi$ during all considered calculations. These variations are smaller for pSic1$^3$ with 0.12$^{\circ }$ and 0.03$^{\circ }$ for $\phi$ et $\psi$, and even smaller for the mixed pools of conformations with 0.06$^{\circ }$ and 0.02$^{\circ }$. Among six of the seven sets of TAiBP conformations (marked in bold), already repeatedly selected by BioEn, were also selected by RamaMix (Tables 1, 2, 3 and S5, S6).

Similarly to the populations obtained by BioEn between the different SAXS data, the populations found using RamaMix are quite different than the ones determined by BioEn. Another difference between BioEn and RamaMix processing is the smaller number of conformations selected by RamaMix, it can arise from the essential difference between the data, as the SAXS curves describe a global picture of the conformations whereas the Ramachandran maps give a local information. A smaller number of conformations are selected from the sets where more extended conformations were included: this may be due to the important conformational drift induced by the systematic choice of extended conformations during the clustering step (see Supplementary Material section “Clustering of generated conformations”).

In order to compare the conformations selected by BioEn on the three SAXS curves, several curves superimpositions have been realized. The superimposition of SAXS curves reconstructed from the conformations selected from Tables 1 and 2 to the corresponding fitted SAXS curves (Fig. S8) displays a reasonable agreement with $\chi ^2$ in the range 0.6-2.06. These values are larger than the ones given in the Tables 1 and 2, due to the fact that conformations displaying populations smaller than 1% have been removed. Besides, a comparison of all sets of BioEn conformations with all SAXS curves (Table S3) reveals that the conformations and populations determined from the fit of one SAXS curve display $\chi ^2$ values with another SAXS curve going up to 4.42. The variability between the three SAXS curves induces thus a drift between conformations and populations selected from the fit of each curve.

A similar comparison has been performed between the SAXS curves and the conformations and populations determined with RamaMix (Fig. S9). In this comparison, the $\chi ^2$ values are in the range 0.98-4.24 which is similar to what is observed for BioEn selected conformations in Table S3. The variability between the fits to Ramachandran maps and SAXS curves is thus similar to the variability of fit between different SAXS curves.

**Figure 4:**
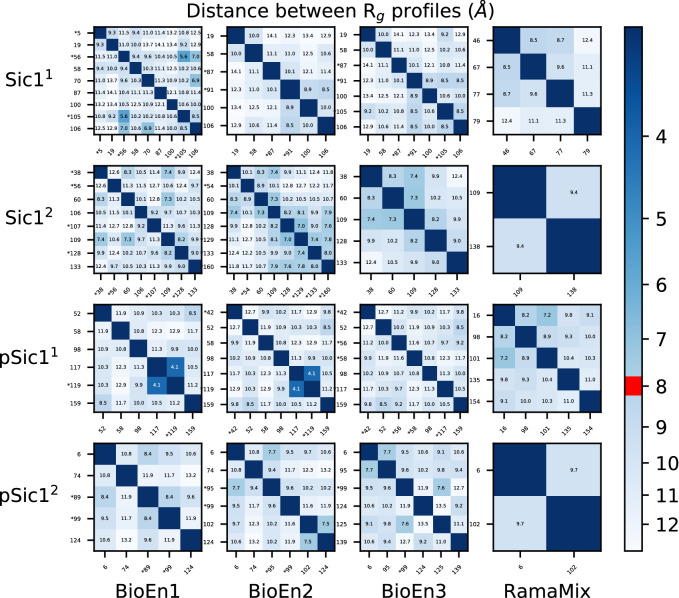
Distances between the profiles $P_q$ (Eq. 1) of local gyration radii between the conformations selected from the fit of SAXS curves (BioEn1,BioEn2, BioEn3) or Ramachandran maps (RamaMix). The conformations for which populations smaller than 10% were calculated, are labeled with an asterisk. The diagonals correspond to the comparison of the same conformations and are thus not annotated with distance value. The limit of 8 Å used to display superimposed plots of profiles $P_q$ (Fig. 5) is drawn in red on the scale of distance.

In order to investigate the possible convergence between the different conformations and populations detected using BioEn and RamaMix, systematic comparison of Euclidean distances between profiles of local gyration $P_q$ (Eq. 1) was performed (Figs. 4, S10 and S11) The Euclidean distances within each set of conformations selected by BioEn reveal (Fig. 4, three left columns) that, for several cases, distances smaller than 8 Å are observed between different conformations. In many cases, such small distances are observed between conformations (labeled with asterisk in Fig. 4) for which populations smaller than 10% are observed. The comparison of profiles $P_q$ (Eq. 1) between conformations selected by RamaMix (Fig. 4, right column) reveals two features. When few conformations have been selected (as for Sic1$^2$ and pSic1$^2$), the distances between their profiles $P_q$ are larger than 8 Å. When more conformations are selected (as for Sic1$^1$ and pSic1$^1$), profile distances smaller than 8 Å are observed. The small $P_q$ distances reveal a certain convergence of the profiles $P_q$.

**Table 1:** Conformations and populations selected using BioEn 0.1.1^18^ on the three sets of SAXS curves. The conformations were generated by the runs Sic1$^1$ and Sic1$^2$. For each SAXS curve and set of protein conformations, after ten runs starting from random values of populations and performed on the whole set of conformations, all conformations for which the sum of populations over the ten runs was larger than 0.01 were gathered, and a second run of ten additional BioEn calculations was performed on this reduced set of conformations. The average and standard deviation values of populations obtained for each selected conformation from the second set of BioEn runs, are given in the Table, along with the final average values of reduced $\chi ^2$ and of entropy $S_{KL}$. The labels of conformations selected in at least two runs are written in bold. The conformations displaying average populations smaller than 1% were removed from the final set.

A. Sic1$^1$	Conformation numbers	Populations percentages	Conformation numbers	Populations percentages	Conformation numbers	Populations percentages
	**100**	15.5 ± 2.2	**100**	19.8 ± 0.2	**100**	21.7 ± 0.1
	**105**	1.4 ± 2.7	**106**	15.0 ± 1.6	**105**	5.0 ± 0.1
	**106**	13.0 ± 1.7	**19**	25.6 ± 0.2	**106**	18.2 ± 0.6
	**19**	18.3 ± 2.2	**58**	24.4 ± 0.4	**19**	21.8 ± 0.1
	56	6.7 ± 3.4	**87**	9.5 ± 0.3	**58**	23.9 ± 0.3
	**58**	4.4 ± 2.2	**91**	5.5 ± 1.8	**87**	7.0 ± 0.0
	5	6.4 ± 2.6			**91**	2.3 ± 0.8
	70	14.6 ± 5.6				
	**87**	19.1 ± 2.8				
Average final $\chi$2		0.4		0.4		0.3
Average final S$_{KL}$		−1.7e-9		−7.9e-10		−5.0e-10

B. Sic1$^2$	Conformation numbers	Populations percentages	Conformation numbers	Populations percentages	Conformation numbers	Populations percentages
	106	17.2 ± 1.0	**38**	13.7 ± 4.6	**109**	43.6 ± 1.3
	107	7.1 ± 0.3	**109**	41.7 ± 7.6	**128**	15.2 ± 1.5
	**109**	27.1 ± 0.9	**128**	12.5 ± 1.4	**133**	10.4 ± 3.5
	**128**	6.6 ± 0.5	129	9.1 ± 3.0	**38**	19.9 ± 0.7
	**133**	17.3 ± 1.2	**133**	1.2 ± 3.5	**60**	10.4 ± 0.2
	**38**	6.9 ± 0.3	160	1.3 ± 3.3		
	56	5.7 ± 0.3	54	3.0 ± 4.9		
	**60**	11.7 ± 0.3	**60**	16.1 ± 1.1		
Average final $\chi$2		0.4		0.4		0.3
Average final S$_{KL}$		−1.0e-8		−1.7e-8		−3.1e-9

**Table 2:** Conformations and populations selected using BioEn 0.1.1^18^ on the three sets of SAXS curves. The conformations were generated by the runs pSic1$^1$ and pSic1$^2$. The Table caption is the same than for Table 1.

A. pSic1$^1$	Conformation numbers	Populations percentages	Conformation numbers	Populations percentages	Conformation numbers	Populations percentages
	**117**	16.1 ± 0.6	**117**	21.2 ± 0.6	**117**	9.1 ± 4.6
	**119**	2.7 ± 0.9	**119**	2.1 ± 1.3	**159**	35.9 ± 3.6
	**159**	32.1 ± 0.1	**159**	22.2 ± 0.6	**42**	7.3 ± 7.6
	**52**	18.9 ± 0.4	**42**	4.4 ± 2.5	**52**	11.0 ± 4.3
	**58**	16.9 ± 0.5	**52**	10.9 ± 0.9	56	2.2 ± 4.3
	**98**	13.2 ± 0.3	**58**	23.0 ± 0.9	**58**	8.1 ± 4.1
			**98**	16.2 ± 1.6	**98**	26.4 ± 3.4
Average final $\chi$2		0.9		1.1		0.7
Average final S$_{KL}$		−1.9e-9		−2.5e-9	−2.3e-10	

B. pSic1$^2$	Conformation numbers	Populations percentages	Conformation numbers	Populations percentages	Conformation numbers	Populations percentages
	**124**	42.3 ± 0.6	102	13.9 ± 7.4	**124**	24.8 ± 1.6
	**6**	39.7 ± 0.4	**124**	37.1 ± 8.4	125	10.2 ± 3.4
	**74**	13.4 ± 0.2	**6**	30.3 ± 4.3	139	30.5 ± 1.3
	89	2.0 ± 0.3	**74**	13.8 ± 0.7	**6**	13.1 ± 0.7
	**99**	2.1 ± 0.7	**95**	1.3 ± 3.6	**95**	20.2 ± 1.2
			**99**	3.6 ± 2.5	**99**	1.2 ± 1.8
Average final $\chi$2		0.8	0.9		0.7	
Average final S$_{KL}$		−3.8e-9		−1.8e-8		−3.8e-10

**Table 3:** Conformations and populations selected by fitting of the Ramachandran maps using RamaMix. For each set of protein conformations, 100 runs were performed starting from random values for the populations. The few converged optimizations which did not converge, were discarded: 6 for Sic1$^1$, 2 for Sic1$^2$, 3 for pSic1$^1$ and 3 for pSic1$^2$. The backbone angles $\phi$ and $\psi$ were allowed to move up to 15$^{\circ }$. The populations of conformations for the converged runs were averaged and these mean values are given as percentages in the Table along with the corresponding standard deviation values. The labels of conformations also selected by BioEn are written in bold.

A. Sic1$^1$	Conformation numbers	Populations percentages
	79	44.7 ± 0.5
	77	23.4 ± 0.6
	67	21.7 ± 0.4
	46	10.2 ± 0.4

B. Sic1$^2$	Conformation numbers	Populations percentages
	**109**	67.8 ± 2.9
	138	32.1 ± 0.8

C. pSic1$^1$	Conformation numbers	Populations percentages
	**98**	23.2 ± 1.4
	154	22.7 ± 2.0
	101	21.2 ± 0.7
	135	19.2 ± 3.3
	16	13.7 ± 1.0

D. pSic1$^2$	Conformation numbers	Populations percentages
	**6**	59.2 ± 3.7
	**102**	40.7 ± 3.0

The comparison of conformations selected by BioEn and RamaMix as well as the comparison between conformations selected from the fit of the various SAXS curves is displayed in Figs. S10 and S11. A close inspection of these distance matrices for BioEn conformations (Fig. S10) shows that, if one excludes the conformations populated less than 10%, there are only three conformations displaying profile distances larger than 8 Å and selected in two distinct BioEn runs: (i) for Sic1$^2$, the conformation 106 selected on the SAXS curve BioEn1 compared to the conformations selected on the two other SAXS curves; (ii) for pSic1$^2$, the conformation 74, selected in the runs BioEn1 and BioEn2, and compared to the conformations selected from the run BioEn3; (iii) for pSic1$^2$, the conformation 139 selected from the run BioEn3, and compared to the conformations from the run BioEn1. Overall, most of the conformations populated more than 10% from the fitting of different SAXS curves display profile distances smaller than 8 Å, supporting a convergence of the profiles in the different fits.

On the other hand, the comparison between BioEn and RamaMix fitting (Fig. S11) displays contrasted behaviors between the duplicated TAiBP runs. For Sic1$^2$ and pSic1$^2$, all RamaMix conformations display profiles closer than 8 Å to the profiles of BioEn conformations. For pSic1$^1$, this is also the case for three RamaMix conformations (16, 98, 101) over five. For Sic1$^1$, only the conformation 79 displays profile distances smaller than 8 Å for the three comparisons.

**Figure 5:**
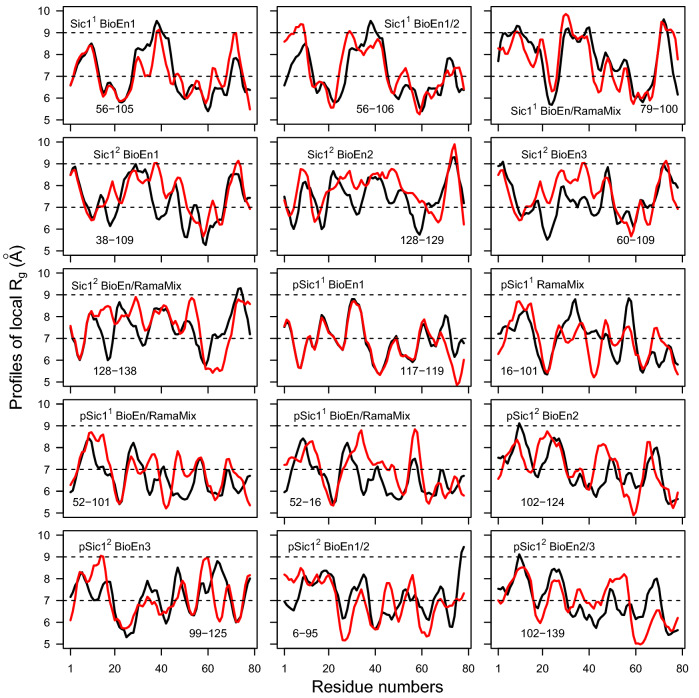
Superposition of profiles $P_q$ (Eq. 1) displaying distances smaller than 8 Å extracted from Figs. 4 and S8, S9. The name of the run (Sic1$^1$, Sic1$^2$, pSic1$^1$, pSic1$^2$) is given, along with the name of the considered fits (BioEn1, BioEn2, BioEn3, RamaMix, RamaMix/BioEn) and the conformations numbers. The labels RamaMix/BioEn correspond to the comparison of conformations selected by BioEn on one side and RamaMix on the other side. The labels BioEn1/2 and BioEn2/3 correspond to the comparison of conformations selected by BioEn from two different SAXS curves.

Examples of profiles $P_q$ superimposition have been chosen accordingly to the values of their distances (Fig. 5) and give an estimation of the connection between the information related to atomic coordinates and the distance between the profiles. These examples represent distances in the 4.05–7.88 Å range. The examination of Fig. 5 reveals that the profile peaks are mostly located at similar places in the protein sequence. This gives a qualitative description of the conformations separated in extended regions (profile maxima) and in aggregated regions (profile minima).

The description of IDP conformations by $P_q$ profiles permits to detect some convergence between the various Bioen fits and also between RamaMix and BioEn fit. This is extremely encouraging due to the enormous conformational size and to the heterogeneity of the measurements (SAXS, NMR) used for fitting the populations. Nevertheless, this comparison remains extremely qualitative, and far from any high resolution description. It could represent a starting point for deeper investigation of IDP conformations.

### Comparison with PED conformations and link with biological activity

The sets of Sic1 and pSic1 conformations selected from the fitting of SAXS curves and of Ramachandran probability maps, were compared to the sets of protein conformations deposited in the Protein Ensemble Database protein ensemble.org^21^.

The values of the resulting gyration radii were calculated (Table 4) from the populations determined by BioEn and RamaMix, and using the individual gyration radii of selected conformations. Globally, the resulting gyration radii display orders of values agreeing with the measurements reported in Fig. 2E of Ref. ^17^. For the conformations extracted from the data-sets Sic1$^1$ and Sic1$^2$, the resulting gyration radii agree with the measurement of 3.0 ± 4.1 Å given in Fig. 2E of Ref. ^17^. But, for pSic1$^1$ and pSic1$^2$, the resulting gyration radii are smaller than the measurements of Ref. ^17^: this is particularly true for the BioEn processing, while the RamaMix processing displays values closer to those of Gomes et al^17^. On the more extended conformations (pSic1$^3$), all resulting gyration radii are significantly closer to Gomes et al^17^ measurements, for BioEn and RamaMix processing (Table 4). Pooling pSic1$^3$ with the conformations of pSic1$^1$ or pSic1$^2$ (sets pSic1$^{13}$ and pSic1$^{23}$) produces different effects for BioEn and RamaMix processing. For these mixed data-sets, the resulting gyration radii obtained from the BioEn processing (range 27.2-28.1 Å) decrease to reach a level just slightly larger than the one obtained for pSic1$^1$ and pSic1$^2$ (range 26.1-27.9 Å). On the contrary, the gyration radii obtained by RamaMix processing are the same than the ones obtained for pSic1$^3$. Overall, the BioEn processing is more sensitive than RamaMix to the presence of conformations with lower gyration radii. The discrepancy of the results obtained here on pSic1 with those shown in Ref. ^17^ arises in part from a tendency to obtain smaller gyration radii by processing of the whole SAXS curve with respect to the larger gyration radii obtained by using the Guinier approximation within the low-q region of the SAXS data.

**Figure 6:**
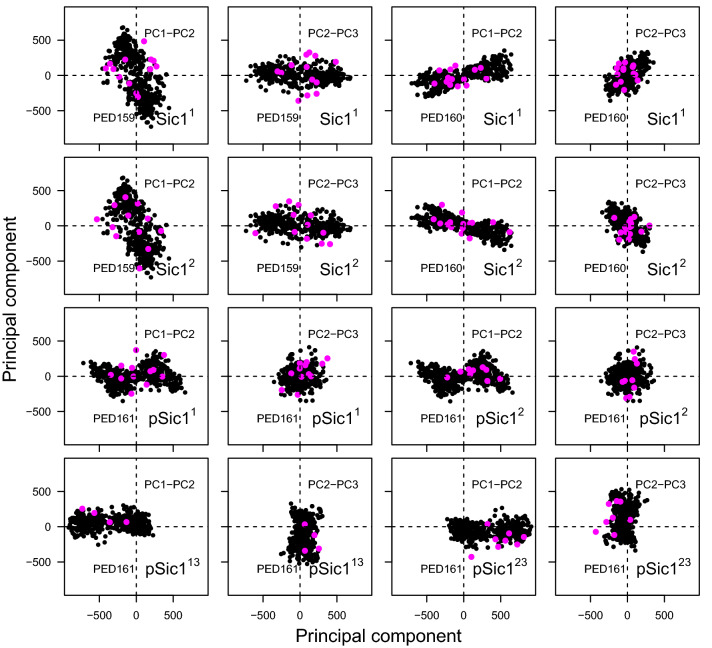
Projections of the Sic1 and pSic1 conformations along the three largest components of their principal component analysis (PCA). On these projections, the TAiBP conformations selected by BioEn or RamaMix are colored in magenta and the conformations stored in PED^21^ are colored in black.

The selected TAiBP conformations were also compared to the PED conformations by realizing a principal component analysis (PCA) of the atomic coordinates. The coordinates projected on the first and second or on the second and third component (Fig. 6) reveal that most of the TAiBP conformations are located in similar space regions than the PED conformations.

**Table 4:** Resulting gyration radii (Å) calculated from the individual gyration radii of the conformations selected by the BioEn and RamaMix analyses. The data-sets Sic1$^1$, Sic1$^2$ and pSic1$^1$, pSic1$^2$, pSic1$^3$ were obtained using the approach TAiBP on the proteins Sic1 and pSic1. The data-sets pSic1$^{13}$ and pSic1$^{23}$ were obtained by pooling together the conformations of pSic1$^1$ and pSic1$^{3}$ or the conformations of pSic1$^2$ and pSic1$^{3}$.

Data-set	BioEn1	BioEn2	BioEn3	RamaMix
Sic1$^1$	27.8	28.7	28.5	31.3
Sic1$^2$	27.7	28.4	28.4	27.1
pSic1$^1$	26.7	26.1	27.2	28.0
pSic1$^2$	27.4	27.1	27.9	30.0
pSic1$^3$	30.4	30.6	30.5	32.4
pSic1$^{13}$	27.4	27.2	28.0	32.5
pSic1$^{23}$	27.4	27.4	28.1	32.5

The presence of phosphorylated residues decreases obviously the global charge of pSic1 with respect to Sic1. It was pointed out that the induced variation in long-range electrostatic interactions plays a role in the electrostatic interaction of pSic1 with its target Cdc14^22^. However, the variations of charges also gives various opportunities for the formation of hydrogen bonds, which were analyzed for the whole set of conformations from the TAiBP runs as well as for the PED sets of conformations. All PED conformations were submitted to the same refinement than the one used on TAiBP conformations described in the Section “Molecular dynamics refinement in implicit solvent” of the Supplementary Material, using positional restraints on protein backbone atoms with a constant force of 50 kcal/mol. The cumulative variations of $\psi$ and $\phi$ angles during the refinement were in the range 0.8-1.2$^{\circ }$ for $\phi$ and in the range 0.3-0.8$^{\circ }$ for $\psi$, and the coordinate RMSD around 0.1 Å. All hydrogen bonds were detected on the refined PED conformations as well as on the TAiBP conformations. Cumulative contact maps (Fig. S12) display these hydrogen bonds, according to the involved residues, the hydrogen bonds involving phosphorylated residues being colored in magenta. The inspection of these contact maps reveals that the PED and TAiBP conformations display distinct tendencies. Long range hydrogen bonds involving phosphorylated residues are more present in the less extended set of TAiBP conformations pSic1$^1$ and pSic1$^2$ than in pSic1$^3$. On the other hand, the conformation set PED161 of pSic1 displays the largest number of long-range hydrogen bonds involving the sidechains of phosphorylated residues. Thus, the presence of phosphorylated residues can induce the appearance of long-range hydrogen bonds whatever is the variation of resulting gyration radius.

## Discussion

The TAiBP approach enumerating the protein conformations in the frame of the distance geometry problem has been used for describing the conformational space of two IDPs, Sic1 and pSic1, corresponding to the unphosphorylated and phosphorylated states of a disordered region involved in the control of S phase in the cellular cycle. The present study represents a test for a new approach able to systematically enumerate protein conformations in the frame of a distance geometry approach. Indeed, up to now, most of the approaches for calculating IDP conformations are based on Monte Carlo approaches^8,23,24^ which do not guarantee an exhaustive exploration of the conformational space.

One should notice that TAiBP overcome the exponential complexity of the branch-and-prune algorithm, due to the parallel calculations on fragments, to the rejection of too close solutions, and to the systematic use of clustering. A major advantage of this approach is the availability of a systematic procedure. Nevertheless, the obtained conformations are only representative conformations, and the represented conformational space has still to be defined.

The use of TAiBP approach permits to avoid the question of the convergence of solutions for protein conformations. The introduction of the profiles of local gyration radii $P_q$ along with their relative populations allows the reintroduction of a convergence criterion into the problem, and this is essential for validation purposes. In the present work, the validity of this convergence criterion has been assessed by the comparison of the profiles $P_q$ obtained from independent fits. In that frame, the profile of local gyration radii could be proposed for describing the IDP conformational space: the knowledge, even qualitative, of the profiles should provide geometrical restraints allowing a more precise exploration of the conformational space. The profiles are closer between the conformations selected by the various fits of SAXS curves, than between the conformations selected by BioEn and RamaMix. This is expected as the various fits of SAXS curves use homogeneous information. More surprisingly, similar profiles are observed between conformations selected by RamaMix and BioEn, for the runs Sic1$^2$ and pSic1$^2$, and for many conformations of the runs Sic1$^1$ and pSic1$^1$.

One specific advantage of the mixture method RamaMix for determining populations of conformations from the likelihood Ramachandran maps is that it has a larger domain of applicability that the BioEn method based on the SAXS curves. Indeed, polydispersity in protein solutions can make difficult to extract conformational information from the SAXS curve. In addition, the chemical shifts from which the likelihood Ramachandran maps are extracted, can be measured in solution as well as in in-cell NMR or for a sequence inserted in a larger protein^25^.

The comparison of the resulting gyration radii obtained from the BioEn and RamaMix processing with the values measured in Ref. ^17^ showed (Table 4) that various ranges of gyration radii are obtained, depending on the clustering procedure in TAiBP, as well as on the method for SAXS processing. In particular, the processing of the whole SAXS curve with BioEn displays a tendency to underestimate the gyration value with respect to the processing of the Guinier curve. The determination of populations from the Ramachandran probability maps, using RamaMix, seems to be less prone to the underestimation of the gyration radius.

The discrepancy between resulting gyration radii obtained by processing the whole SAXS curve (BioEn) or restricting the analysis to the low-q region (Guinier approximation^17^) agrees with independent calculations performed using coarse-grained protein model^26^, in which various distribution of gyration values produce very similar SAXS spectra (Fig. 10a of Ref. ^26^) or different disordered ensembles produce similar Kratky plots (Fig. 13 of Ref. ^26^).

## Methods

### Origins of the data

Three sets of conformations for Sic1 and pSic1 were available from the Protein Ensemble Database (PED) proteinensemble.org^21^: PED159 and PED160 for pSic1 and PED161 for Sic1^17^. The residue numbering used here is the one proposed in the PED. The NMR chemical shifts were downloaded from the Biological Magnetic Resonance Data Bank (BMRB)^27^ as entries: 16657 for Sic1^17^ and 16659 for pSic1^19^. The SAXS data-sets recorded as triplicate sets in the conditions described in Ref. ^17^ were provided by Tanja Mittag.

### Enumeration of conformations using TAiBP

The protein conformations have been enumerated using the recently proposed TAiBP approach^10–12^, which generalizes the interval branch-and-prune (iBP) algorithm^9,28–31^ so as to overcome the combinatorial barrier arising from the enormous space of IDP conformations^32^. TAiBP is composed of two steps: (i) the enumeration of conformations for peptide fragments (Table S1) spanning the studied protein using individual iBP calculations; (ii) the enumeration of Sic1 and pSic1 conformations by systematic assembly of fragment conformations in a way similar to what is used in the field of protein prediction^33^.

The boxes of backbone angles $\phi$ and $\psi$ used as inputs for the iBP step were determined from the Ramachandran likelihood maps predicted by TALOS-N^14^ (see section “Extraction of boxes from Ramachandran likelihood maps” and Figs. S1–S4 in Supplementary Material). The $\phi$/$\psi$ boxes were systematically combined by permutation to prepare individual iBP calculations as in Ref^11^. The enumeration of conformations is realized by the building of a tree, each node of the tree corresponding to an atomic position. The tree building allows the enumeration of the various possibilities for atom positions (branching step) whereas additional geometric information is used to accept or reject a newly built branch (pruning step). As the angles $\phi$ and $\psi$ are straightforwardly related to distances between atoms C and N of residues successive in the sequence^11,12^, the discretization of intervals of these angles is used in the branching step. In the iBP step, the pruning was applied by preventing atoms to be closer than the sum of their van der Waals radii and by checking that the improper angle values are correct. In addition, each solution displaying a coordinate root-mean-square deviation (RMSD) smaller than 2 Å with the previously stored solution, is rejected. The details of the iBP step calculation are described in the section “Enumeration of conformations” of the Supplementary Material.

The assembly step is also performed with a branch-and-prune approach using as elementary blocks, not the atoms, but the fragment conformations previously determined during the iBP step. Two peptide fragments are assembled by superimposing the three last and initial residues of the fragments successive in the protein sequence. The fragments are then merged in the following way: the atom at which the smallest distance was observed between corresponding atoms in the two peptides was used to decide where to stop with the first peptide and to continue with the second one. The assembled conformations in which C$\alpha$ atoms closer than 1 Å are observed, were pruned from the calculation. The fragment assembly was implemented using python scripting based on the MDAnalysis^34,35^ and numpy 1.7.1^36^ packages.

To scale down the combinatorial explosion of the calculation, a clustering approach based on **S**elf-**O**rganizing **M**aps (SOM)^37–40^ was systematically applied to the generated sets of conformations larger than 100 during the iBP and assembly steps. The details of this approach are described in the section “Clustering of generated conformations” of the Supplementary Material.

After the assembly step, the sidechains have been added to the conformation backbones, and the conformations were refined by molecular dynamics simulations as described in the section ”Molecular dynamics refinement in implicit solvent” in the Supplementary Material.

### Determining the population from Ramachandran maps

The approach RamaMix, based on a finite mixture model, was designed to determine the populations of conformations by fitting on the Ramachandran probability maps. The setting-up of this approach is based on the hypothesis that the likelihood maps describing the likelihood of the TALOS-N prediction^14^ can be transformed by normalization into the probability density of the presence of $\phi$ and $\psi$ values in the set of conformations populated in solution.

Consequently, for each residue *n*, the Ramachandran probability map is denoted as a 2D probability density $p^n(\phi ,\psi )$, modeled as a mixture of probability densities $p_q^n(\phi ,\psi )$ determined on each conformation *q*:

2 $$\begin{aligned} p^n(\phi ,\psi ) = \sum _{q=1}^{Q} \gamma _{q} p_{q}^n(\phi ,\psi ) \end{aligned}$$

where $\gamma _q \ge 0$ is the population of conformation *q* in solution.

RamaMix intends to decompose the probability map $p^n(\phi ,\psi )$ according to Eq. (2) along the following lines: (i) the total number *Q* of conformations is taken from output of TAiBP; (ii) for each conformation *q* and each residue *n*, $p_{q}^n(\phi ,\psi )$ is a periodized Gaussian density characterized by averaged values of backbone angles $(\phi ^n_q, \psi ^n_q)$ and by a $2\times 2$ covariance matrix $C^n_q$; (iii) the populations $\gamma _q$ have to be adjusted in order to maximize the fit between the Ramachandran probability maps and the mixture model (Eq. 2).

The Ramachandran probability maps $p^n(\phi ,\psi )$ are jointly fitted to the finite mixture model (Eq. 2) using a discrepancy measure between both probability maps given by the Kullback-Leibler divergence:

3 $$\begin{aligned} D_{KL}(p_1||p_2)=\int p_1(x)\ln \frac{p_1(x)}{p_2(x)} dx. \end{aligned}$$

Calculations detailed in the Supplementary material (Sections “Determination of the populations from the Ramachandran maps” and “Maximum likelihood estimation for bivariate sine mixtures”) show that using the Kullback-Leibler divergence is equivalent to the maximization of the log-likelihood of the data^41^:

4 $$\begin{aligned} \mathcal {L}(y;\theta ) = \sum _{n=1}^{N}\sum _{m=1}^{M} \ln p^n(\phi _m,\psi _m). \end{aligned}$$

For the sake of clarity, let us first introduce a standard, non-periodized Gaussian density $p_{q}^n(\phi ,\psi )$ for the residue *n* in conformation *q*:

5 $$\begin{aligned} p_{q}^n(\phi ,\psi ) = \frac{1}{2\pi } \det (C^n_q)^{-1/2} \exp ({- V^n_q(\phi ,\psi )}) \end{aligned}$$

where $V^n_q(\phi ,\psi )$ represents the free energy surface for the basin around the conformation *q*. The free energy surface is described in the frame of an elastic network model on the backbone dihedral angles^42–45^:

6 $$\begin{aligned} V^n_q(\phi ,\psi ) = \frac{1}{2} \theta _q^{t} [C^n_q]^{-1} \theta _q \end{aligned}$$

where: $\theta _q = (\phi -\phi ^n_q, \psi -\psi ^n_q)^{t}$, $\phi ^n_q$ and $\psi ^n_q$ are the values of dihedral angles of the residue *n* in the conformation *q* and $C^n_q$ is the corresponding covariance. The software IMOD^42^ was used for determining the full Hessian (*N*, *N*) (*N* is the total number of residues in the protein) matrix $H_q$ along the backbone dihedral angles. The Hessian matrix is then inverted to produce: $C_q = H_q^{-1}$. The covariance matrix $C^n_q$ of the angles $\phi$ and $\psi$ of the considered residue *n* is the (2,2) sub-matrix of $C_q$, centered on the two $\phi _q^n$ and $\psi _q^n$ angles. The inverse of this matrix $[C^n_q]^{-1}$ is used in Eq. (6).

As the protein conformations are described by couples of angles, we must consider that the support of the probability densities $p_{q}^n(\phi ,\psi )$ is a torus, i.e., that they are doubly circular. Following^46–48^, we replaced Eq. (6) by a bivariate extension of the von Mises distribution, as being more easily tractable than a Gaussian density wrapped on the torus. More precisely, we adopt a bivariate periodic sine model^46^:

7 $$\begin{aligned} p(\phi ,\psi ) = \frac{1}{T} \exp ({W(\phi -\phi _0,\psi -\psi _0)}) \end{aligned}$$

with

8 $$\begin{aligned} W(\phi ,\psi ) = \kappa _1\cos \phi +\kappa _2\cos \psi +\lambda \sin \phi \sin \psi , \end{aligned}$$

$\kappa _1,\kappa _2\ge 0$ and $\lambda ^2<\kappa _1\kappa _2$. According to Ref. ^46^, the integration constant *T* is expressed as an infinite series, depending on parameters $(\kappa _1,\kappa _2,\lambda )$:

9 $$\begin{aligned} T=4\pi ^2\sum _{m=0}^\infty \left( {\begin{array}{c}2m\\ m\end{array}}\right) \left( \frac{\lambda ^2}{4\kappa _1\kappa _2}\right) ^m I_m(\kappa _1)I_m(\kappa _2) \end{aligned}$$

where $I_m$ denotes the modified Bessel functions of the first kind of order *m*^49^.

In Ref. ^46^, expressions of $(\kappa _1, \kappa _2, \lambda )$ are given as functions of the parameters $(\sigma _1^2,\sigma _2^2,\rho )$ of a bivariate Gaussian where $\rho \in (-1,1)$ denotes the normalized correlation coefficient between the two components of the bivariate Gaussian:

10 $$\begin{aligned} \sigma _1^2=\frac{\kappa _2}{\kappa _1\kappa _2-\lambda ^2},\quad \sigma _2^2=\frac{\kappa _1}{\kappa _1\kappa _2-\lambda ^2},\quad \rho =\frac{\lambda }{\sqrt{\kappa _1\kappa _2}}. \end{aligned}$$

These expressions are valid only in the case where $\sigma _1^2$ and $\sigma _2^2$ are small. They are easily inverted as

11 $$\begin{aligned} \kappa _1=\frac{1}{\sigma _1^2}\frac{1}{1-\rho ^2},\quad \kappa _2=\frac{1}{\sigma _2^2}\frac{1}{1-\rho ^2},\quad \lambda =\frac{1}{\sigma _1\sigma _2}\frac{\rho }{1-\rho ^2}. \end{aligned}$$

Using (11), we can replace a Gaussian mode $p_{q}^n$ by a periodized version, with approximately the same location and the same spread. In the following, we will describe basin shapes around conformations using the triplets of parameters $(\kappa _1, \kappa _2, \rho )$ rather than $(\kappa _1, \kappa _2, \lambda )$, since $\rho ^2<1$ is a simpler constraint than its counterpart on $\lambda$.

A well-known local optimization scheme to identify finite mixture models by maximum likelihood is the Expectation-Maximization (EM) algorithm^50,51^. Unfortunately, the M step of the EM has no analytical expression in the case of mixtures of bivariate Von-Mises densities. Therefore, we have performed local optimization based on L-BFGS-B^52^ instead, given that both the likelihood and its gradient can be evaluated efficiently, and that some parameters are subject to box constraints. The implementation details and equations are given in the sections “Determination of the populations from the Ramachandran maps” and “Maximum likelihood estimation for bivariate sine mixtures” of the Supplementary Material.

By optimization of the log-likelihood, the RamaMix approach will thus produce the *Q* normalized populations $\gamma _q$, the $Q\times N$ couples of backbone angles $\phi _q^n$ and $\phi _q^n$, as well as the $Q\times N$ triplets $(\kappa _1^n, \kappa _2, \rho _q^n)$ describing the von Mises distributions. The calculations were performed starting from the $\phi$ and $\psi$ values observed in the set of TAiBP conformations, complemented by von Mises parameters allowing us to approximate the Gaussian distributions determined by IMOD. Moreover, the variation of $\phi$ and $\psi$ values was limited by a threshold of 15$^{\circ }$ during the optimization in order to avoid inappropriate drift.

The RamaMix approach was implemented in Fortran90, and the software is available at github.com/tmalliavin/RamaMix.

### Determining the populations from SAXS data

The software BioEn 0.1.1^18^ was used in order to determine the populations from SAXS data. On each considered conformation, theoretical SAXS curves were calculated using CRYSOL^53^ available in the package ATSAS 3.0.3^54^ with 847 points, a maximum scattering vector of 0.503 nm$^{-1}$ and a maximum order of harmonics of 18. A 1D cubic interpolation^55^ was used to obtained the theoretical SAXS values at the same sets of scattering vectors *q* than the ones at which the experimental SAXS curve was recorded.

The processing with BioEn was performed in the following way. For each TAiBP run and each SAXS curve, the optimization was run for 1000 steps using the GSL library^56^. Ten runs were performed independently on all considered conformations, and the subset of conformations for which the sum of observed populations is larger than 0.01, was selected. Ten additional BioEn runs were performed on the subset of conformations, and from the results of these ten repetitions, average values and standard deviations were computed for the populations.

## Supplementary Information

Supplementary Information.

## Data Availability

The datasets used for producing the Figures of the main text as well as the generated IDP conformations are available at: 10.5281/zenodo.7198645. Other datasets used or/analyzed during the current study are available from the corresponding author on reasonable request.
