# Supplementary material for: Low-resolution description of the conformational space for intrinsically disordered proteins
Source: Sci Rep. 2022 Nov 9;12:19057. doi: 10.1038/s41598-022-21648-9 (PMC9646904; doi:10.1038/s41598-022-21648-9)
Supplement: Supplementary file 1 — Supplementary Information. [file 41598_2022_21648_MOESM1_ESM.pdf]

Supplementary Material: Low-resolution description of the conformational space for intrinsically disordered proteins

Daniel Förster (1), Jérôme Idier (2), Leo Liberti (3), Antonio Mucherino (4), Jung-Hsin Lin (5) and Thérèse E. Malliavin (6,7,8)

(1) UMR7374 Interfaces, Confinement, Matériaux et Nanostructures, Université d'Orléans, France

(2) UMR6004 Laboratoire des Sciences du Numérique de Nantes, France

(3) LIX UMR 7161 CNRS École Polytechnique, Institut Polytechnique de Paris, 91128 Palaiseau, France

(4) IRISA, University of Rennes 1, France

(5) Biomedical Translation Research Center, Academia Sinica, Taiwan

(6) Institut Pasteur, Université Paris Cité, CNRS UMR3528, Unité de Bioinformatique Structurale, F-75015 Paris, France

(7) Laboratoire de Physique et Chimie Théoriques (LPCT), University of Lorraine, Vandoeuvre-lès-Nancy, France

(8) Laboratoire International Associé, CNRS and University of Illinois at Urbana-Champaign, Vandoeuvre-lès-Nancy, France

Corresponding authors:

Thérèse E. Malliavin, therese.malliavin@univ-lorraine.fr

Jérôme Idier, jerome.idier@ls2n.fr

Short title

Conformational space of IDPs Sic1 and pSic1

September 5, 2022

Extraction of boxes from Ramachandran likelihood

The likelihood Ramachandran maps, calculated by TALOS-N [1], were first normalized in order to get the sum of values equal to 1 and to produce probability maps. Each ϕ, ψ box was determined from these maps in the following way. In the current state of the Ramachandran map, the pixels belonging to a box are removed from the map. From the remaining pixels, the pixel of maximum probability value and larger than the threshold, is selected and a box is iteratively drawn around this position by testing systematically all pixels neighboring the current box limits. All neighbouring pixels containing values larger than a given threshold are included in the box. If values are smaller than the threshold, the calculation stops, the current box definition is kept for further analyses and the pixels selected from the box are removed from the Ramachandran map. This approach is iteratively applied to the map up to the situation where all remaining map pixels display values smaller than the threshold. In order to probe the reproducibility of TAiBP results, two sets of boxes have been determined with threshold values of 0.01 (Figures S1 and S3) and 0.011 (Figures S2 and S4).

In pSic1, the presence of phosphorylated Threonines 7, 35 and 47 and of phosphorylated Serines 71, 78 and 82 makes impossible the TALOS-N predictions for residues 5-9, 33-37,

45-49, 69-73, 76-84, due to the lack of phosphorylated proteins in the learning set of the neural network. Thus, for these residues, generic boxes (Table S2) have been used as input of TAiBP, in order to cover the Ramachandran regions corresponding to α-helix, extended, β strand and loop structures.

Enumeration of conformations

The enumeration of protein conformations was performed using boxes of backbone angles ϕ and ψ. These boxes (Figures S1-S4) have been extracted from the likelihood Ramachandran maps obtained by TALOS-N [1] as described in the previous section. During the tree building, each atomic position is determined by trilateration from the previously determined atomic positions, following a specific ordering (Table S4) [2]. More precisely, two out of three of the distances involved in trilateration must be known exactly, and one may be subject to uncertainty and represented by an interval [2, 3]. The iBP algorithm was the one described by Worley et al [4, 3].

The backbone dihedral angles ϕ and ψ can be straightforwardly related to bond lengths and bond angles and respectively to distances between atoms C of residues $i-1$ and i and between atoms N of residues i and $i+1$. This equivalence between the backbone dihedral angles and inter-atomic distances permits to use the angles ϕ and ψ for the so-called branching step. This branching step is performed by discretization of the intervals in order to generate new branches in the tree.

The bond lengths, bond angles, improper angles and van der Waals radii were taken from the force field protein-allhdg5-4 PARALLHDG (version 5.3) [5, 6]. The van der Waals radii

were scaled by a factor of 0.7.

For each fragment, two dummy residues were added at the N and C terminal extremities, and the ϕ and ψ dihedral angles of the inner peptide residues were sampled according to the box limits (Table S1). In order to avoid pruning due to slight discrepancy between distances, a tolerance of 0.05 Å has been added to the bounds of distance intervals. The maximum number of branches by discretized interval was set to 4. The minimum discretization factor, which is the minimum ratio between each distance interval to the number of tree branches generated within the interval, was set to 0.1 Å, in order to avoid that the branching over-samples small intervals. A maximum number of 10^9 saved conformations was permitted for each iBP run. The solutions were stored in a multiframe dcd format [7].

Clustering of generated conformations

The approach **S**elf-**O**rganizing **M**ap (SOM) [8, 9, 10, 11], used to cluster conformations, is an artificial neural network (ANN) trained using unsupervised learning. SOM displays the advantage with respect to the k-means clustering approach that it does not require the predetermined knowledge of the number of clusters. The SOM approach was used after each iBP calculation or assembly step as soon as the number of saved conformations was larger than 100. The conformations are encoded from the distances d_{ij} calculated between the n C_α atoms by diagonalizing the covariance matrix C:

$$C_{i,j} = \frac{1}{n}\sum_{k=1}^{n}\sum_{l=1}^{n}(d_{i,k} - \bar{d}_i)(d_{l,j} - \bar{d}_j) \tag{1}$$

where $\bar{d}_s = \frac{1}{n}\sum_{p=1}^{n} d_{s,p}$. The information contained in the matrix C is equivalent to its four largest eigenvalues along with the corresponding eigenvectors, and is formatted as an

input vector of length $4(n+1)$. These vectors are used to train a periodic Euclidean 2D self-organizing map (SOM), which corresponds to a three-dimensional matrix. The first two matrix dimensions were chosen to be 100×100 and define the map size, the third dimension being equal to $4(n+1)$. Each vector along the third dimension defines a neuron of the map. The neurons of the self-organizing map are initialized with a random uniform distribution covering the range of values of the input vectors. At each step, an input vector is presented to the map, and the neurons closest to this input are updated. The training parameters were those previously described [12, 11].

Once the SOM has been determined, representative conformations are extracted from the conventional **U**nified **d**istance **m**atrix (U-matrix) calculated from the final SOM neurons. For each neuron ν, the corresponding U-matrix element is calculated as the average Euclidean distance between the neuron ν and its eight immediate neighbors:

$$\text{U-matrix}(\nu) = \frac{1}{8} \sum_{\nu \in N(\nu)} d(\nu, \mu) \quad (2)$$

where $N(\nu)$ is the set of neighbors, and $d(\nu, \mu)$ is the Euclidean distance between the neurons μ and ν. pSic1^3 The neurons corresponding to local minima of the U-matrix, and thus to local maxima of conformational homogeneity, are extracted and for all performed runs except pSic1^3, the protein conformation displaying the closest distance to each neuron is saved. In the case of pSic1^3, among the conformations saved in the neurons, the one displaying the longest distance between the Carbons α of the first and the last residues is saved, in order to obtain more extended conformations in agreement with the values of gyration radii measured in Ref. [13]. The conformations generated during the iBP or assembly steps are finally replaced by the sets of representative conformations extracted from local minima of

U-matrix.

Molecular dynamics refinement in implicit solvent

Molecular dynamics (MD) trajectories were used to relax the Sic1 and pSic1 conformations obtained from the TAiBP approach. The MD trajectories were recorded using NAMD 2.13 [14]. Topology parameters were taken from the force fields c36 [15] and c36m [16]. The simulations were performed at a temperature of 300 K. A Generalized Born implicit solvent (GBIS) [17] model was used with an ion concentration of 0.3M, and a cutoff of 12 Å for calculating Born radius. A cutoff of 14 Å and a switching distance of 13 Å were defined for non-bonded interactions. The RATTLE algorithm [18, 19] was used to keep all covalent bonds involving hydrogens rigid, enabling a time step of 2 fs. Temperature was regulated according to a Langevin thermostat [20]. At the beginning of each trajectory, the system was first minimized for 1,000 steps, then heated up gradually from 0 K to 300 K in 30,000 integration steps. Finally, the system was equilibrated for 5,000 steps. During all steps, from minimization to production, positional restraints were applied on protein backbone atoms with a constant force of 1 kcal/mol. A production run of 100ps was then performed and the conformation of the final frame was saved as the relaxed conformation.

Determination of the populations from the Ramachandran maps

Using the neural network TALOS-N [1], it is possible, starting from the NMR chemical shifts measured on protein atoms, to determine for each residue n a 2D probability density $p^n_{\text{TALOS-N}}(\phi, \psi)$. As NMR analyses a sample containing a mixture of conformations, we propose to decompose the probability map produced by TALOS-N as a mixture of probability densities $p^n_q(\phi, \psi)$, corresponding to a certain number of free energy basins q present in the experimental sample:

$$p^n_{\text{TALOS-N}}(\phi, \psi) \approx \sum_{q=1}^{Q} \gamma_q p^n_q(\phi, \psi) \tag{3}$$

where $\gamma_q \geq 0$ is the proportion of local basins q in the NMR sample. Thus:

$$\sum_{q=1}^{Q} \gamma_q = 1. \tag{4}$$

In the following, the problem described by Eq. 3 will be named as a Q-class mixture problem, each class corresponding to a conformation of the studied protein. In addition, for each conformation q, the couple of angles corresponding to the bottom of the basin will be named its location parameters.

To fit the set of N available TALOS-N probability densities using the mixture model (3), we have to rely on a discrepancy measure between both probability maps. Kullback-Leibler divergence is a standard choice:

$$D_{\text{KL}}(p_1 || p_2) = \int p_1(x) \ln \frac{p_1(x)}{p_2(x)} dx. \tag{5}$$

Here, we consider the sum of discrepancy measures over the N residues between TALOS-N

probability densities and the corresponding mixture model densities $p^n = \sum_{q=1}^{Q} \gamma_q p_q^n$:

$$\sum_{n=1}^{N} D_{\mathrm{KL}}(p^n_{\text{TALOS-N}} \,||\, p^n) = \sum_{n=1}^{N} \int p^n_{\text{TALOS-N}}(\phi,\psi) \ln \frac{p^n_{\text{TALOS-N}}(\phi,\psi)}{p^n(\phi,\psi)} d\phi d\psi. \tag{6}$$

In practice, TALOS-N probability densities are available on a finite rectangular grid $\{\phi_1, \ldots, \phi_I\} \times \{\psi_1, \ldots, \psi_J\}$. Let us modify (6) using a zeroth-order approximation of the integrals:

$$\sum_{n=1}^{N} D_{\mathrm{KL}}(p^n_{\text{TALOS-N}} \,||\, p_2) = \sum_{n=1}^{N} \sum_{i=1}^{I} \sum_{j=1}^{J} \hat{p}^n_{ij} \ln \frac{\hat{p}^n_{ij}}{p^n_{ij}}. \tag{7}$$

where $\hat{p}^n_{ij} = p^n_{\text{TALOS-N}}(\phi_i, \psi_j)$ and

$$p^n_{ij} = p^n(\phi_i, \psi_j) = \sum_{q=1}^{Q} \gamma_q p^n_q(\phi_i, \psi_j). \tag{8}$$

Finally, the minimization of Eq. 7 with respect to $\gamma = (\gamma_1, \ldots, \gamma_Q)$ amounts to the maximization of

$$f(\gamma) = \sum_{n=1}^{N} \sum_{i=1}^{I} \sum_{j=1}^{J} \hat{p}^n_{ij} \ln p^n_{ij}, \tag{9}$$

$p^n_{ij}(\phi, \psi)$ being given by (8), under the constraints $\gamma_q \geq 0$ and $\sum_{q=1}^{Q} \gamma_q = 1$. Let us remark that f is a concave function of γ, so that its local maximization with respect to γ cannot be trapped in a local maximum.

In case TALOS-N yielded observations in the form of angle couples $y^n_m = (\phi^n_m, \psi^n_m)$, $m = 1, \ldots, M$, instead of a probability map, we would naturally maximize the log-likelihood of the data [21],

$$\mathcal{L}(y; \theta) = \sum_{n=1}^{N} \sum_{m=1}^{M} L^n(\phi^n_m, \psi^n_m), \tag{10}$$

a well-known local optimization scheme to reach this goal being the EM algorithm [22, 23]. Let us remark the similarity between Eqs. (9) and (10). In fact, Eq. (9) identifies with the log-likelihood of virtual data, each couple (ϕ_i, ψ_j) being observed $D^n_{ij} = \hat{p}^n_{ij} \times C$ times

for the nth residual (C being an arbitrary constant). This corresponds to a well-known correspondance between the log-likelihood and the Kullback-Leibler divergence between the empirical data distribution and the parametrized one (see for instance [23]).

In the case where data points correspond to couples of angles, we must consider that the support of densities p_q is a torus, i.e., that they are doubly circular. The most natural circular extension of the univariate Gaussian is the wrapped normal distribution. However, the von Mises distribution is usually considered as a better option, being more easily tractable [24]. Moreover, multivariate extensions exist for the latter. In particular, in the Ref. [25] a bivariate version was introduced, motivated by problems of modelling torsional angles in molecules, and a pseudo-maximum likelihood method was proposed [24] to estimate its parameters. Moreover, a so-called *cosine* version was investigated [26] and an Expectation-Maximization (EM) algorithm was used [26, 27] to solve a problem that is almost identical to ours.

Here, we adopt the same bivariate periodic sine model as [25]:

$$p(\phi, \psi) = \frac{1}{T} \exp(W(\phi - \phi_0, \psi - \psi_0)) \tag{11}$$

with

$$W(\phi, \psi) = \kappa_1 \cos \phi + \kappa_2 \cos \psi + \lambda \sin \phi \sin \psi \tag{12}$$

and $\kappa_1, \kappa_2 \geq 0$ and $\lambda^2 < \kappa_1 \kappa_2$. A difficulty is that the integration constant is expressed as an infinite series, depending on parameters $(\kappa_1, \kappa_2, \lambda)$:

$$T = 4\pi^2 \sum_{m=0}^{\infty} \binom{2m}{m} \left(\frac{\lambda^2}{4\kappa_1 \kappa_2} \right)^m I_m(\kappa_1) I_m(\kappa_2). \tag{13}$$

In Ref. [25], expressions of κ_1, κ_2, λ are given as functions of the parameters $(\sigma_1^2, \sigma_2^2, \rho)$

of a bivariate Gaussian:

$$\sigma_1^2 = \frac{\kappa_2}{\kappa_1\kappa_2 - \lambda^2}, \quad \sigma_2^2 = \frac{\kappa_1}{\kappa_1\kappa_2 - \lambda^2}, \quad \rho = \frac{\lambda}{\sqrt{\kappa_1\kappa_2}}. \tag{14}$$

where $\rho \in (-1, 1)$ denotes the normalized correlation coefficient between the two components of the bivariate Gaussian. These expressions are valid only in the case where σ_1^2 and σ_2^2 are small. They are easily inverted as

$$\kappa_1 = \frac{1}{\sigma_1^2}\frac{1}{1-\rho^2}, \quad \kappa_2 = \frac{1}{\sigma_2^2}\frac{1}{1-\rho^2}, \quad \lambda = \frac{1}{\sigma_1\sigma_2}\frac{\rho}{1-\rho^2}. \tag{15}$$

Using (15), we can replace a Gaussian mode p_q^n by a periodized version, with approximately the same location and the same spread. This is not specific to the Gaussian case, so it also holds for the bivariate von Mises-type model.

In the following section "Maximum likelihood estimation for bivariate sine mixtures", we are deriving the equations describing an original approach for solving the problem (3) by a maximum likelihood approach.

Maximum likelihood estimation for bivariate sine mixtures

Let $Y = (y_1, \ldots, y_D)$ stand for D iid datapoints. We make the assumption that each y_d is sampled from a Q-class mixture model, and we use the notation C_d to refer to the random class attached to y_d, taking values in $(1, \ldots, Q)$. For each d, we have

$$p(y_d; \theta) = \sum_{q=1}^{Q} \Pr(C_d = c_q)\, p(y_d | C_d = c_q; \zeta) = \sum_{q=1}^{Q} \gamma_q\, p(y_d; \zeta_q^{\mathrm{L}}, \zeta_q^{\mathrm{S}}) \tag{16}$$

with unknown parameters $\theta = (\gamma, \zeta) = (\gamma, \zeta^{\mathrm{L}}, \zeta^{\mathrm{S}})$, including

- normalized weights $\gamma = (\gamma_q)$,
- location parameters $\zeta^{\mathrm{L}} = (\zeta^{\mathrm{L}}_q)$ where $\zeta^{\mathrm{L}}_q = (\phi_q, \psi_q)$ is specific to class q,
- shape parameters $\zeta^{\mathrm{S}} = (\zeta^{\mathrm{S}}_q)$ where $\zeta^{\mathrm{S}}_q = (\kappa_{1q}, \kappa_{2q}, \lambda_q)$ is specific to class q.

We would like to estimate θ according to the maximum likelihood principle:

$$\hat{\theta} = \arg\max_{\theta} p(Y; \theta).$$

where $p(Y;\theta) = \prod_{d=1}^{D} p(y_d; \theta)$. Equivalently, $\hat{\theta}$ maximizes the log-likelihood, which reads

$$L(Y;\theta) = -\sum_{d=1}^{D} \ln\left(\sum_{q=1}^{Q} \gamma_q\, p(y_d; \zeta^{\mathrm{L}}_q, \zeta^{\mathrm{S}}_q)\right).$$

In the following, we are first describing what should be an Expectation-Maximization (EM) algorithm adapted for solving the maximum likelihood problem, to finally remark that the Maximization step of the EM cannot be solved analytically. Thus, we turn to a solution based on a well-grounded gradient-based optimization scheme. We derive explicit expressions for the gradient terms, on the basis of the Expectation step of the EM. At the end of this section, we present how to include into the optimization scheme, several Ramachandran probability maps corresponding to several protein residues.

Expectation-Maximization (EM) algorithm

The EM algorithm is a reference solution to determine $\hat{\theta}$ by iterative local optimization. Each EM iteration consists in solving the following auxiliary problem:

$$\theta^{\mathrm{new}} = \arg\max_{\theta} Q(\theta; \theta^{\mathrm{old}}), \tag{17}$$

where Q is the expectation of the log-likelihood of the "complete" dataset:

$$Q(\theta, \theta^{\text{old}}) = \mathrm{E}\left[\ln\left(\Pr(C;\gamma)\, p(Y|C;\zeta)\right)|Y;\theta^{\text{old}}\right] \tag{18}$$

$$= Q_0(\gamma, \theta^{\text{old}}) + Q_1(\zeta, \theta^{\text{old}}) \tag{19}$$

with

$$Q_0(\gamma, \theta^{\text{old}}) = \mathrm{E}\left[\ln \Pr(C;\gamma)|Y;\theta^{\text{old}}\right], \tag{20}$$

$$Q_1(\zeta, \theta^{\text{old}}) = \mathrm{E}\left[\ln p(Y|C;\zeta)|Y;\theta^{\text{old}}\right]. \tag{21}$$

On the one hand, along classical derivations, we get

$$Q_0(\gamma, \theta^{\text{old}}) = \sum_{q=1}^{Q}\left(\sum_{d=1}^{D} P_{qd}\right)\ln\gamma_q \tag{22}$$

where $P_{qd} = P_q(y_d)$, with

$$P_q(y) = \Pr(C = q|y;\theta^{\text{old}}) = \frac{\gamma_q^{\text{old}} p_q(y;\zeta^{\text{old}})}{\sum_{q'}\gamma_{q'}^{\text{old}} p_{q'}(y;\zeta^{\text{old}})}. \tag{23}$$

On the other hand,

$$Q_1(\zeta, \theta^{\text{old}}) = \sum_{d=1}^{D}\sum_{q=1}^{Q} P_{qd}\ln p(y_d;\zeta_q^{\text{L}},\zeta_q^{\text{S}}). \tag{24}$$

The optimization problem (17) splits in two parts at each iteration, according to

$$\gamma^{\text{new}} = \arg\max_{\gamma} Q_0(\gamma, \theta^{\text{old}}), \tag{25}$$

$$\zeta^{\text{new}} = \arg\max_{\zeta} Q_1(\zeta, \theta^{\text{old}}). \tag{26}$$

The first subproblem is constrained by $\sum_q \gamma_q = 1$. It has a simple, explicit solution. Unfortunately, the second subproblem cannot be solved analytically for the sine model, neither for the shape parameters ζ^{S}, nor for the location parameters ζ^{L}. As a consequence, exact

closed-form EM formulas do not exist for the sine model. To our best knowledge, the same holds for other von Mises type models, such as the cosine version of [26]. Indeed, we guess that the EM algorithm used therein solves the maximization step in an approximate way. We rather propose a different solution, relying on a well-grounded gradient-based optimization scheme (namely, the L-BFGS-B algorithm [28]) applied to the log-likelihood itself.

Gradient-based log-likelihood maximization

Fisher's identity [29] relates the gradient of Q to the gradient of the log-likelihood L:

$$\frac{\partial}{\partial\theta}Q(\theta;\theta^{\mathrm{old}})\bigg|_{\theta=\theta^{\mathrm{old}}} = \frac{\partial}{\partial\theta}L(Y;\theta)\bigg|_{\theta=\theta^{\mathrm{old}}} \tag{27}$$

This property is very useful when the M step is not closed-form, since it allows one to replace non-explicit EM iterations by explicit gradient-based iterations, directly applicable to the log-likelihood.

Partial derivative w.r.t. the weights γ

Given Eqs (19), (22) and (27), we have

$$\frac{\partial}{\partial\gamma_q}L(Y;\theta)\bigg|_{\theta=\theta^{\mathrm{old}}} = \frac{\partial}{\partial\gamma_q}Q_0(\gamma,\theta^{\mathrm{old}})\bigg|_{\theta=\theta^{\mathrm{old}}} = \left(\sum_{d=1}^{D}P_{qd}\right)\frac{1}{\gamma_q}. \tag{28}$$

Optimization w.r.t. the weights must be conducted under the constraints of nonnegativity and sum-to-one. The latter can be easily handled using the simple reparameterization $\gamma_q = \frac{\gamma'_q}{\sum_r \gamma'_r}$. It is easy to establish that

$$\frac{\partial}{\partial\gamma'_q}L(Y;\theta)\bigg|_{\theta=\theta^{\mathrm{old}}} = \left(\sum_{d=1}^{D}P_{qd}\right)\frac{1}{\gamma'_q} - \frac{D}{\sum_{r=1}^{Q}\gamma'_r}.$$

Partial derivative w.r.t. the shape parameters ζ^{S}

Given Eqs (19), (24) and (27), we have

$$\frac{\partial}{\partial \zeta_q^{\mathrm{S}}} L(Y;\theta)\bigg|_{\theta=\theta^{\mathrm{old}}} = \frac{\partial}{\partial \zeta_q^{\mathrm{S}}} Q_1(\theta;\theta^{\mathrm{old}})\bigg|_{\theta=\theta^{\mathrm{old}}} = \sum_{d=1}^{D} P_{qd} \frac{\partial}{\partial \zeta_q^{\mathrm{S}}} \ln p(y_d;\zeta_q^{\mathrm{L}},\zeta_q^{\mathrm{S}}) \tag{29}$$

where $p(y_d;\zeta_q^{\mathrm{L}},\zeta_q^{\mathrm{S}})$ is a sine density defined by (11). Explicit expressions for the partial derivative depend on each shape parameter, according to

$$\frac{\partial}{\partial \kappa_1} \ln p(\phi,\psi) = \cos(\phi-\phi_0) - \frac{1}{T}\frac{\partial T}{\partial \kappa_1} \tag{30}$$

$$\frac{\partial}{\partial \kappa_2} \ln p(\phi,\psi) = \cos(\psi-\psi_0) - \frac{1}{T}\frac{\partial T}{\partial \kappa_2} \tag{31}$$

$$\frac{\partial}{\partial \lambda} \ln p(\phi,\psi) = \sin(\phi-\phi_0)\sin(\psi-\psi_0) - \frac{1}{T}\frac{\partial T}{\partial \lambda}, \tag{32}$$

where, given the expression of T (Eq. (13)) and $I'_m(u) = \frac{m}{u} I_m(u) + I_{m+1}(u)$ (see [30]),

$$\frac{\partial T}{\partial \kappa_1} = 4\pi^2 \sum_{m=0}^{\infty} \binom{2m}{m} \left(\frac{\lambda^2}{4\kappa_1\kappa_2}\right)^m I_{m+1}(\kappa_1) I_m(\kappa_2) \tag{33}$$

$$\frac{\partial T}{\partial \kappa_2} = 4\pi^2 \sum_{m=0}^{\infty} \binom{2m}{m} \left(\frac{\lambda^2}{4\kappa_1\kappa_2}\right)^m I_m(\kappa_1) I_{m+1}(\kappa_2) \tag{34}$$

$$\frac{\partial T}{\partial \lambda} = \frac{8\pi^2}{\lambda} \sum_{m=1}^{\infty} \binom{2m}{m} \left(\frac{\lambda^2}{4\kappa_1\kappa_2}\right)^m m I_m(\kappa_1) I_m(\kappa_2). \tag{35}$$

Optimization must be performed under the nonlinear inequality constraint $\lambda^2 < \kappa_1\kappa_2$. A simpler alternative consists in replacing λ by $\rho = \lambda/\sqrt{\kappa_1\kappa_2}$ in the parameterization, so the constraint becomes $\rho \in (-1,1)$. We then need to replace Eq. (12) by

$$W(\phi,\psi) = \kappa_1 \cos\phi + \kappa_2 \cos\psi + \sqrt{\kappa_1\kappa_2}\rho \sin\phi \sin\psi \tag{36}$$

and (30)-(32) by

$$\frac{\partial}{\partial \kappa_1} \ln p(\phi, \psi) = \cos(\phi - \phi_0) + \frac{\lambda}{2\kappa_1} \sin(\phi - \phi_0) \sin(\psi - \psi_0) - \frac{1}{T} \frac{\partial T}{\partial \kappa_1} \tag{37}$$

$$\frac{\partial}{\partial \kappa_2} \ln p(\phi, \psi) = \cos(\psi - \psi_0) + \frac{\lambda}{2\kappa_2} \sin(\phi - \phi_0) \sin(\psi - \psi_0) - \frac{1}{T} \frac{\partial T}{\partial \kappa_2} \tag{38}$$

$$\frac{\partial}{\partial \rho} \ln p(\phi, \psi) = \frac{\lambda}{\rho} \sin(\phi - \phi_0) \sin(\psi - \psi_0) - \frac{1}{T} \frac{\partial T}{\partial \rho}, \tag{39}$$

with

$$\frac{\partial T}{\partial \kappa_1} = 4\pi^2 \sum_{m=0}^{\infty} \binom{2m}{m} \left(\frac{\rho}{2}\right)^{2m} I'_m(\kappa_1) I_m(\kappa_2) \tag{40}$$

$$\frac{\partial T}{\partial \kappa_2} = 4\pi^2 \sum_{m=0}^{\infty} \binom{2m}{m} \left(\frac{\rho}{2}\right)^{2m} I_m(\kappa_1) I'_m(\kappa_2) \tag{41}$$

$$\frac{\partial T}{\partial \rho} = \frac{8\pi^2}{\rho} \sum_{m=1}^{\infty} \binom{2m}{m} \left(\frac{\rho}{2}\right)^{2m} m I_m(\kappa_1) I_m(\kappa_2), \tag{42}$$

given

$$T = 4\pi^2 \sum_{m=0}^{\infty} \binom{2m}{m} \left(\frac{\rho}{2}\right)^{2m} I_m(\kappa_1) I_m(\kappa_2). \tag{43}$$

Partial derivative w.r.t. the location parameters ζ^{L}

Given Eqs (19), (22) and (27), we have

$$\frac{\partial}{\partial \zeta_q^{\mathrm{L}}} L(Y; \theta)\Big|_{\theta=\theta^{\mathrm{old}}} = \frac{\partial}{\partial \zeta_q^{\mathrm{L}}} Q_1(\theta; \theta^{\mathrm{old}})\Big|_{\theta=\theta^{\mathrm{old}}} = \sum_{d=1}^{D} P_{qd} \frac{\partial}{\partial \zeta_q^{\mathrm{L}}} \ln p(y_d; \zeta_q^{\mathrm{L}}, \zeta_q^{\mathrm{S}}). \tag{44}$$

Moreover,

$$\frac{\partial}{\partial \phi_0} \ln p(\phi, \psi) = \kappa_1 \sin(\phi - \phi_0) - \lambda \cos(\phi - \phi_0) \sin(\psi - \psi_0) \tag{45}$$

$$\frac{\partial}{\partial \psi_0} \ln p(\phi, \psi) = \kappa_2 \sin(\psi - \psi_0) - \lambda \sin(\phi - \phi_0) \cos(\psi - \psi_0) \tag{46}$$

Case of multiple datasets

In the case where N residues are available, each conformation is characterized by a unique weight vector, whereas its location and shape parameters are specific to each residue. The identification problem then consists in estimating:

- Q normalized weights $\gamma = (\gamma_q)$ for the protein conformations (classes),
- $5NQ = 3NQ + 2NQ$ shape and location parameters specific to each conformation and each residue, respectively $\zeta_{qn}^{\mathrm{S}} = (\kappa_{1qn}, \kappa_{2qn}, \lambda_{qn})$ and $\zeta_{qn}^{\mathrm{L}} = (\phi_{qn}, \psi_{qn})$.

The log-likelihood then reads

$$L(Y;\theta) = \sum_{n=1}^{N} \sum_{d=1}^{D_n} \ln \left(\sum_{q=1}^{Q} \gamma_q \, p(y_{dn}; \zeta_{qn}^{\mathrm{S}}, \zeta_{qn}^{\mathrm{L}}) \right)$$

where the nth residue corresponds to D_n observed pairs of angles y_{dn}.

The gradient component relative to each shape or location parameter can still be calculated using the equations (37)-(42) and (44)-(46), respectively, while a summation of Eq. (28) over all residues must be performed to obtain the gradient components relative to the weight parameters.

The scheme developed here has been used to calculate the relative weigths of the conformations by fitting the probability Ramachandran maps obtained using TALOS-N [1].

Figures

Figure S1

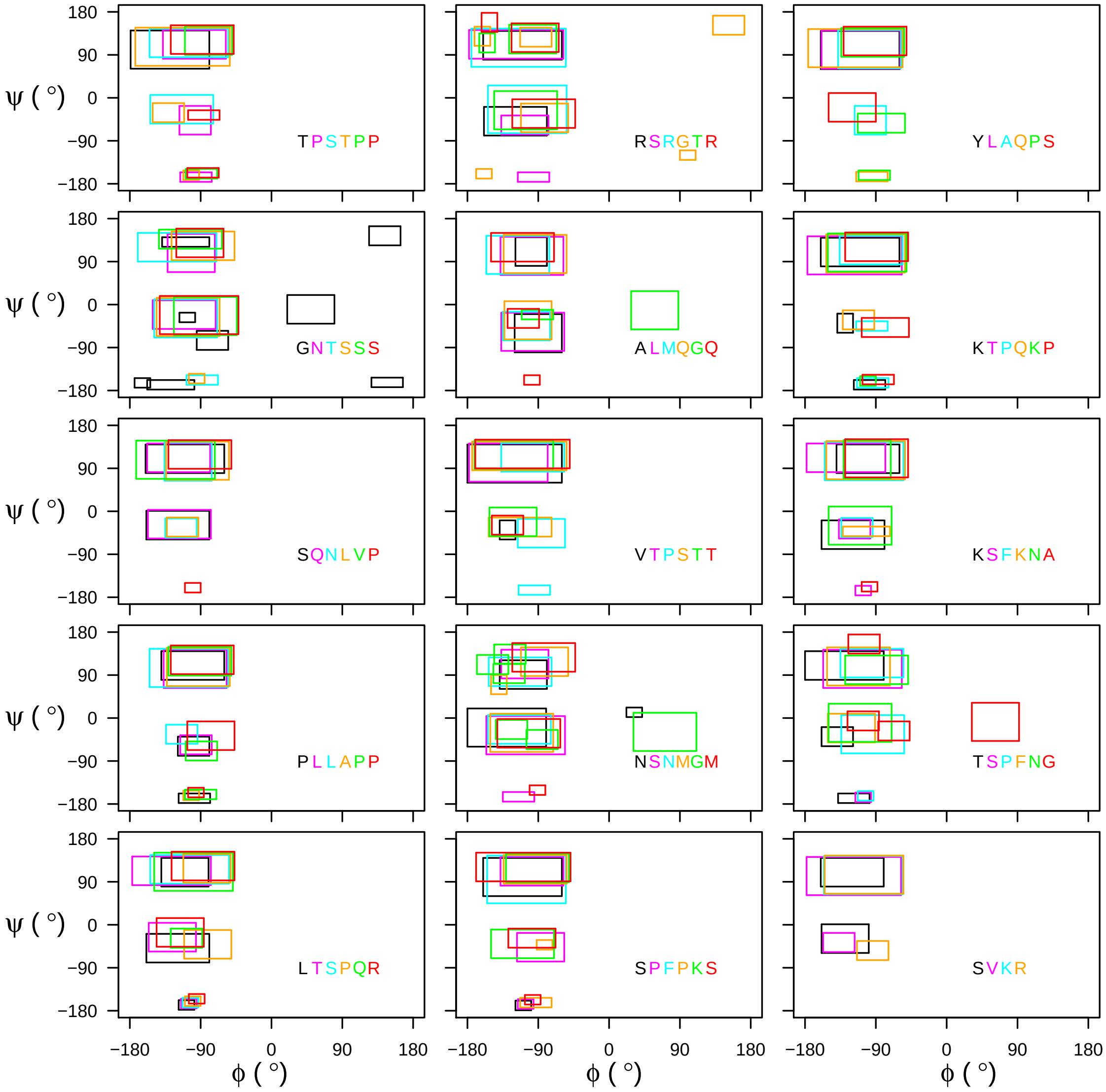

Figure S1: Boxes for backbone angles used as inputs for the run Sic1[1] and obtained from the Ramachandran maps using a threshold of 0.01. The boxes and the corresponding sequence are colored according to the considered residue.

Figure S2

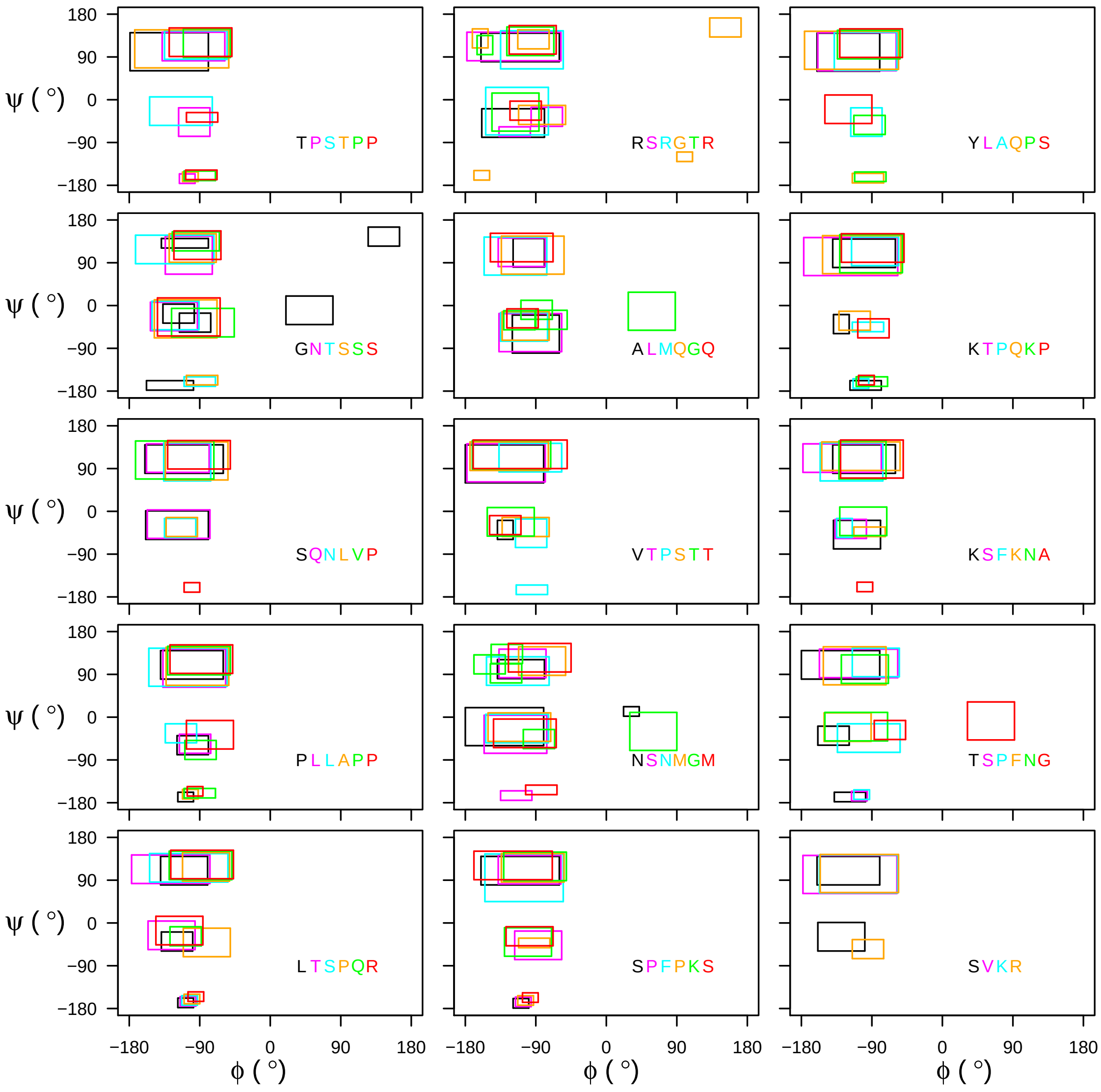

Figure S2: Boxes for backbone angles used as inputs for the run Sic1[2] and obtained from the Ramachandran maps using a threshold of 0.011. The boxes and the corresponding sequence are colored according to the considered residue.

Figure S3

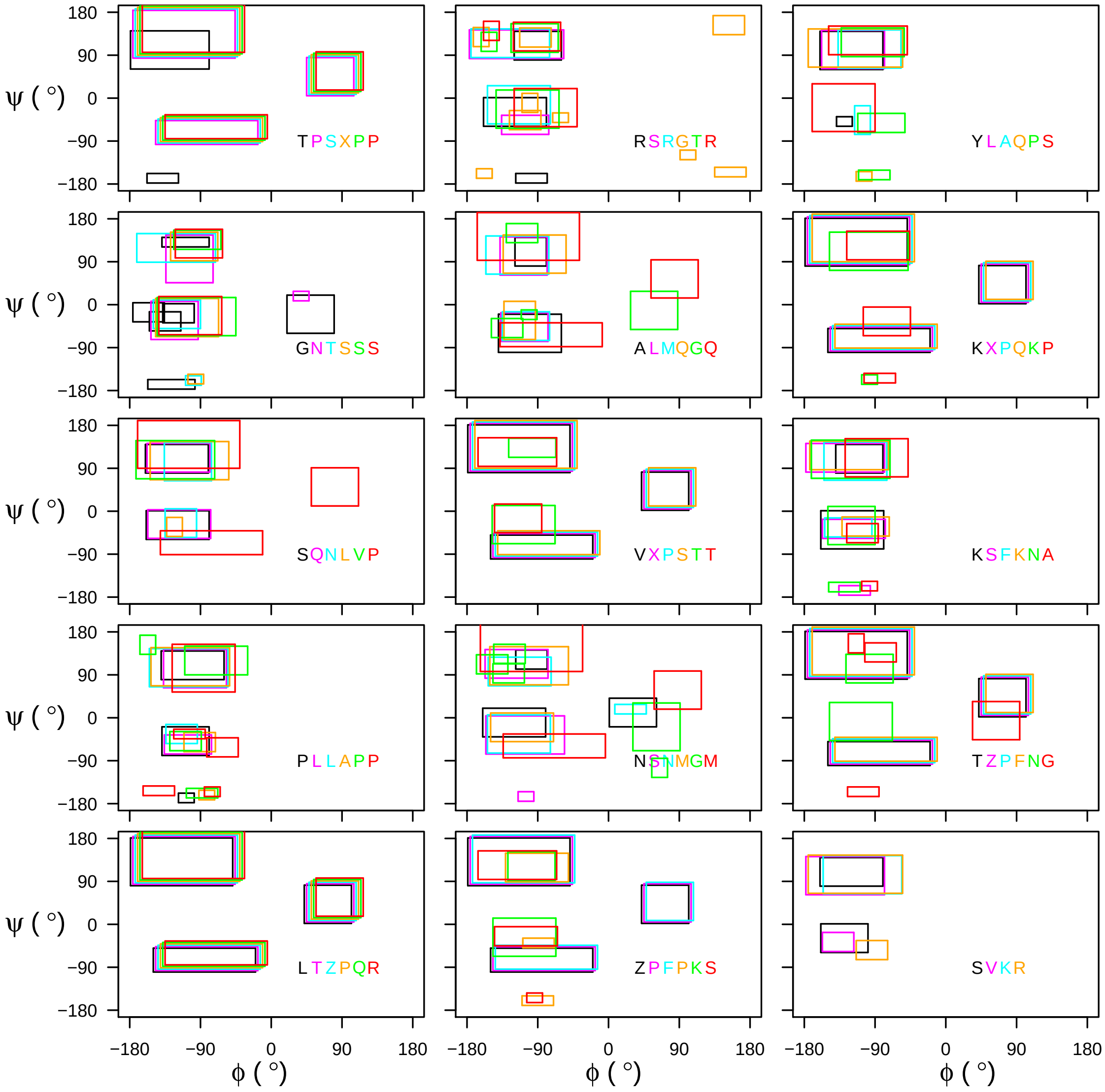

Figure S3: Boxes for backbone angles used as inputs for the run pSic1[1] and obtained from the Ramachandran maps using a threshold of 0.01. The boxes and the corresponding sequence are colored according to the considered residue. The pT and pS residues are marked as X and Z in the sequences.

Figure S4

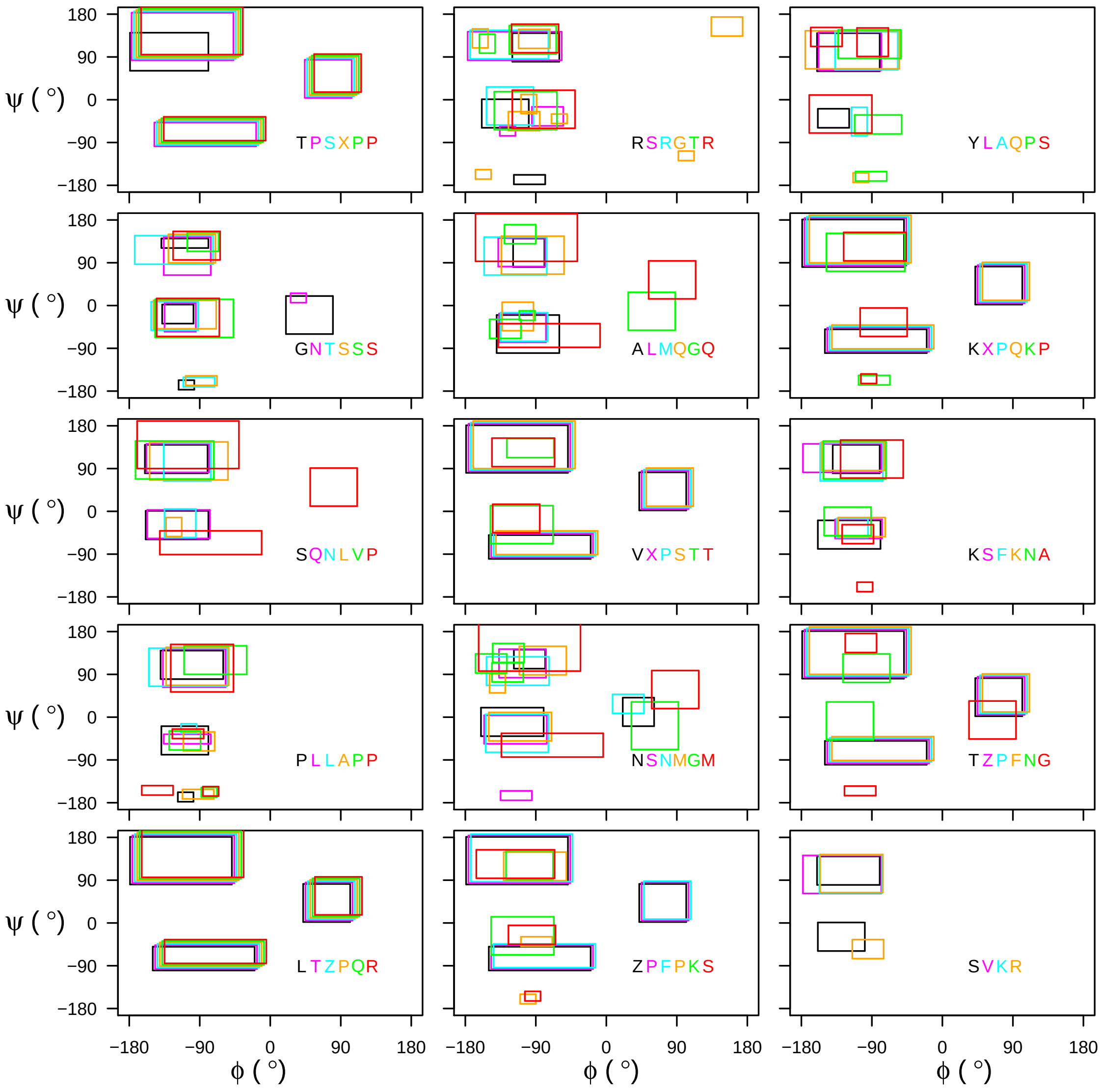

Figure S4: Boxes for backbone angle used as inputs for the run pSic1[2] using a threshold of 0.011. The boxes and the corresponding sequence are colored according to the considered residue. The pT and pS residues are marked as X and Z in the sequences.

Figure S5 (3 next pages)

Figure S5: Superimposition of the MERA ϕ, ψ distributions obtained on residues of Sic1 with the (ϕ, ψ) input boxes for TAiBP. The size of the points on MERA distribution is large for predicted probability values larger than 0.005 and small for the other probability values. The TAiBP input boxes are colored in magenta and green for the duplicated TAiBP runs: $Sic1^1$ and $Sic1^2$.

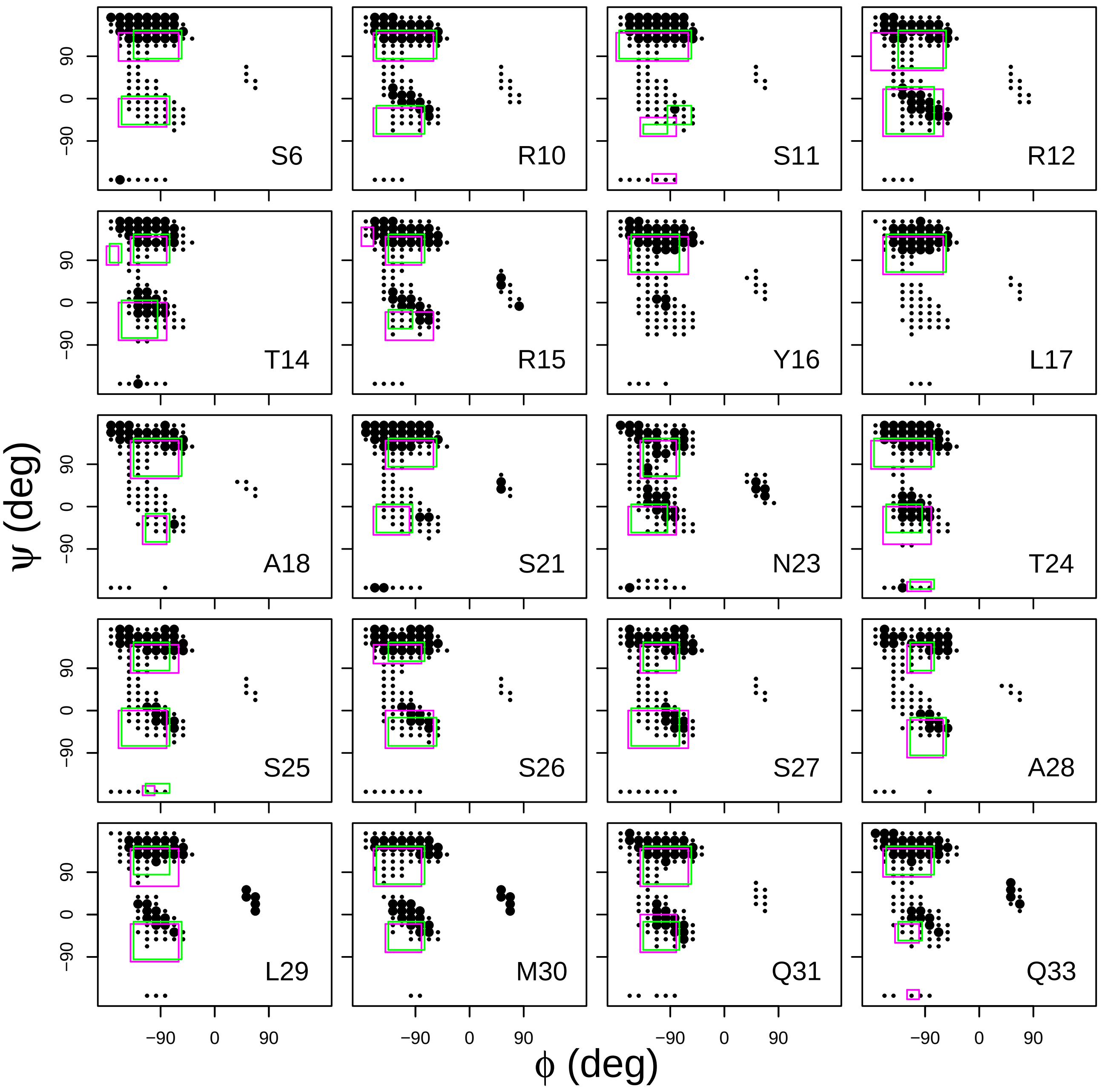

S6
R10
S11
R12
T14
R15
Y16
L17
A18
S21
N23
T24
S25
S26
S27
A28
L29
M30
Q31
Q33
−90
0
90
ψ (deg)
ϕ (deg)

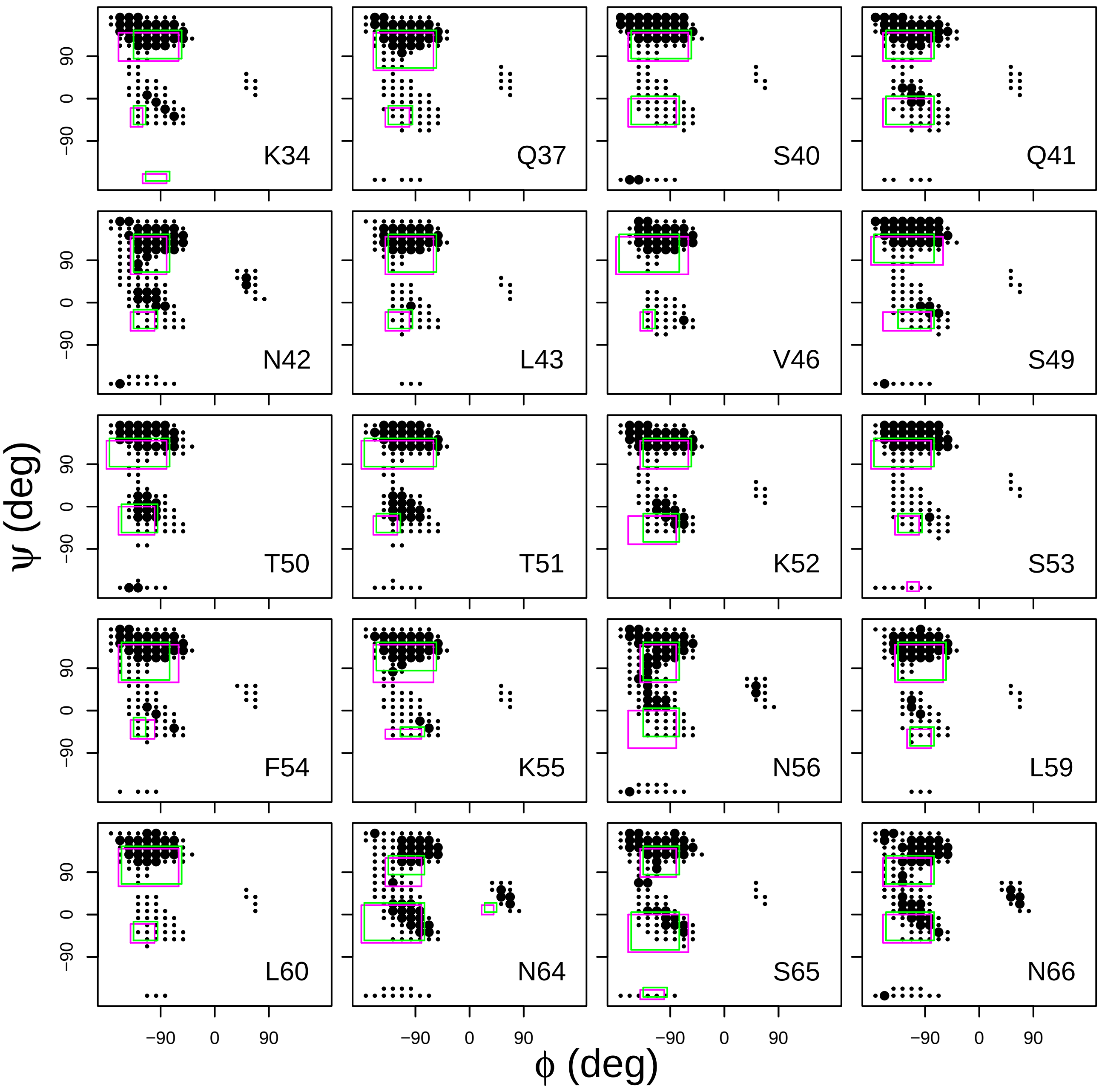
K34
Q37
S40
Q41
N42
L43
V46
S49
T50
T51
K52
S53
F54
K55
N56
L59
L60
N64
S65
N66
90
0
−90
ψ (deg)
−90
0
90
ϕ (deg)

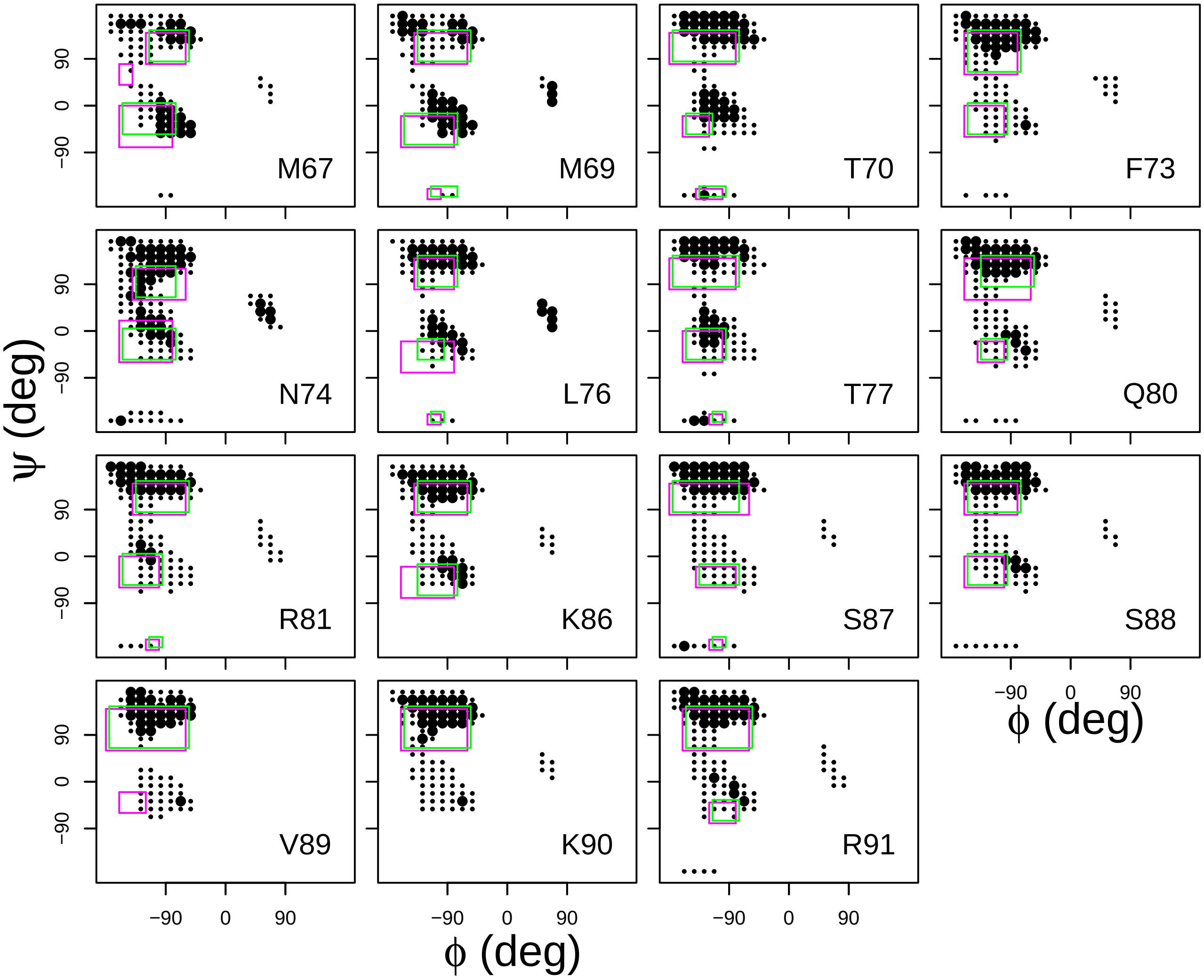

M67
M69
T70
F73
N74
L76
T77
Q80
R81
K86
S87
S88
V89
K90
R91
ψ (deg)
ϕ (deg)
−90
0
90

Figure S6 (3 next pages)

Figure S6: Superimposition of the MERA ϕ, ψ distributions obtained on residues of pSic1 with the (ϕ, ψ) input boxes for TAiBP. The size of the points on MERA distribution is large for predicted probability values larger than 0.005 and small for the other probability values. The TAiBP input boxes are colored in magenta and green for the duplicated TAiBP runs: $pSic1^1$ and $pSic1^2$.

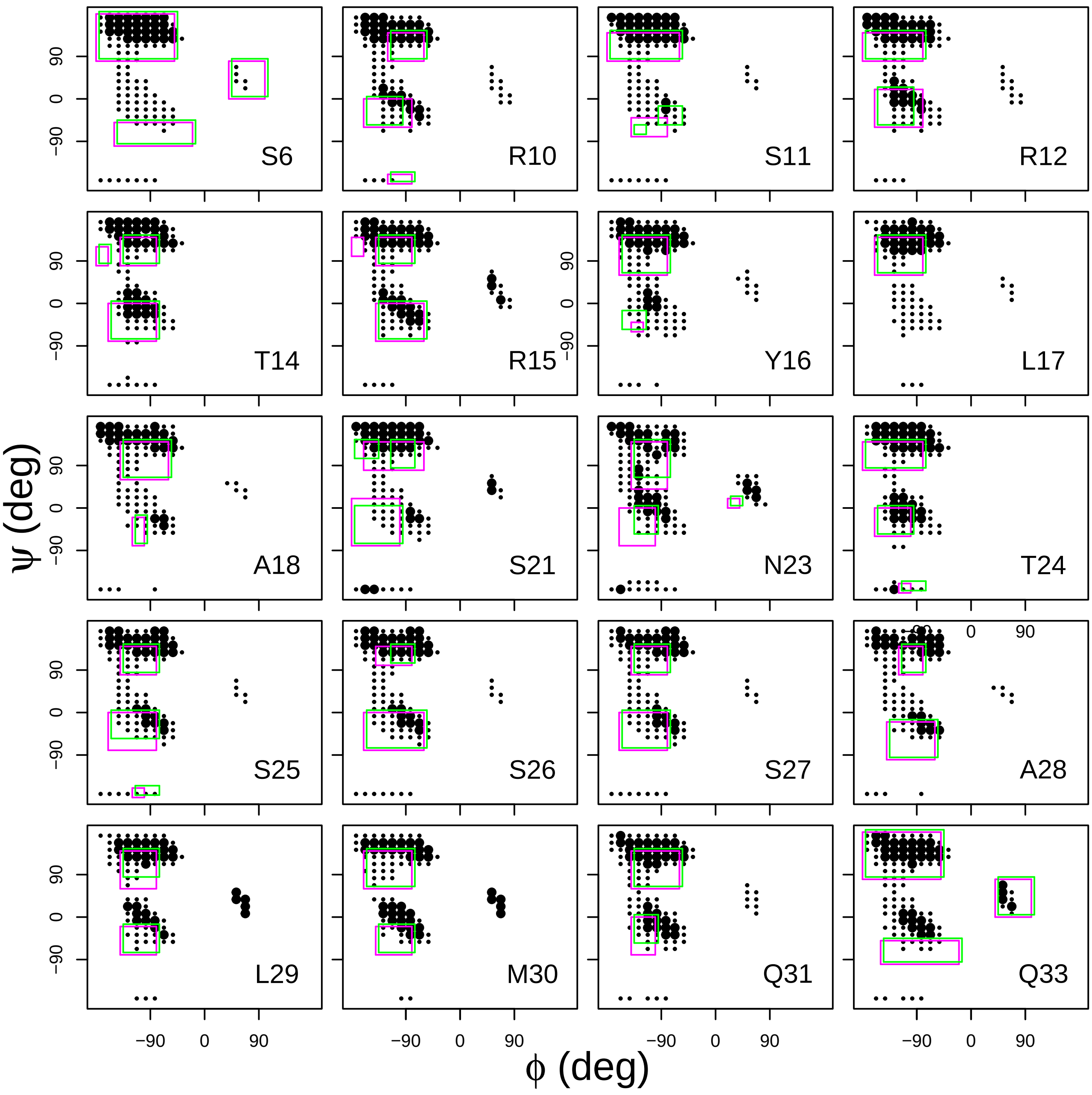
S6
R10
S11
R12
T14
R15
Y16
L17
A18
S21
N23
T24
S25
S26
S27
A28
L29
M30
Q31
Q33
ψ (deg)
ϕ (deg)
−90
0
90

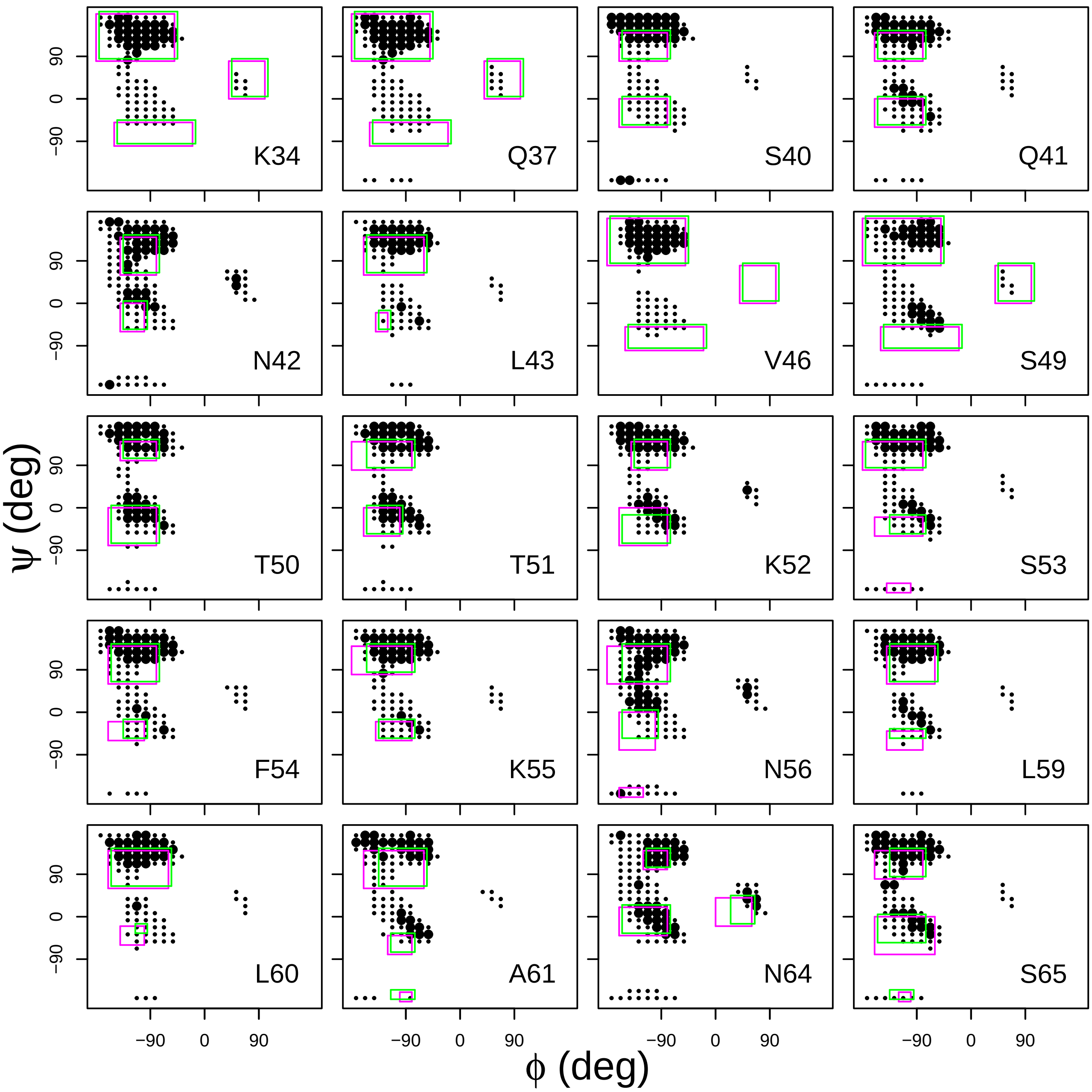

K34
Q37
S40
Q41
N42
L43
V46
S49
T50
T51
K52
S53
F54
K55
N56
L59
L60
A61
N64
S65
ψ (deg)
ϕ (deg)
90
0
−90
−90
0
90

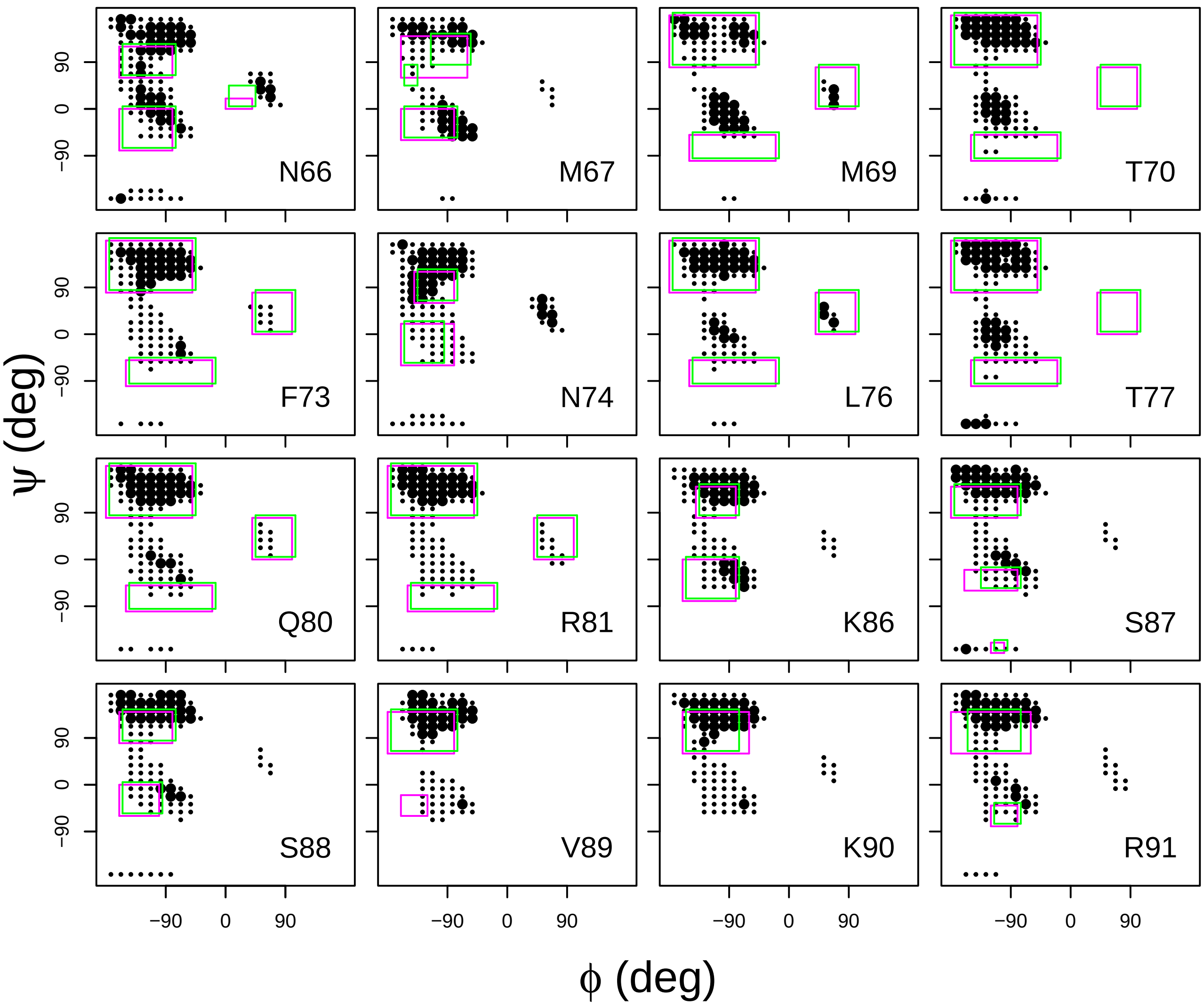

N66
M67
M69
T70
F73
N74
L76
T77
Q80
R81
K86
S87
S88
V89
K90
R91
ψ (deg)
ϕ (deg)
90
0
−90

Figure S7

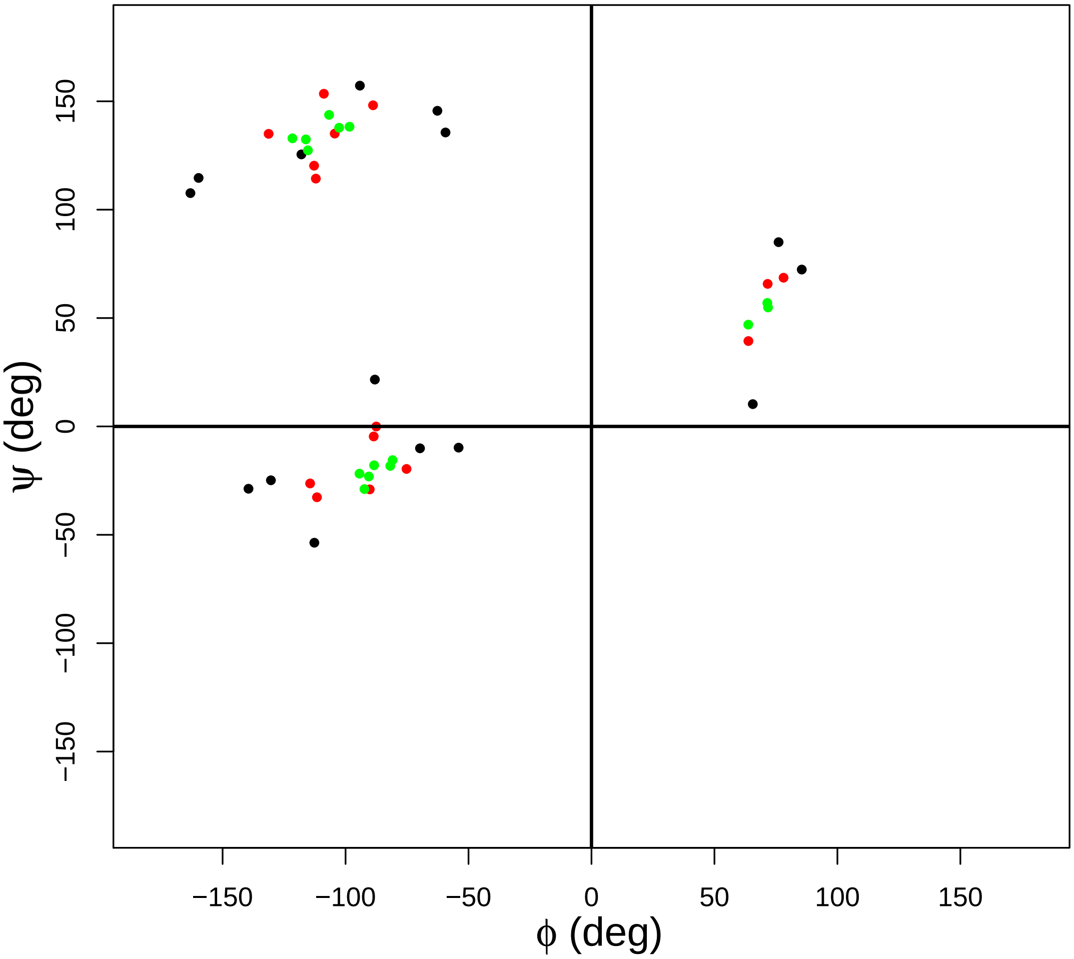

Figure S7: Synthetic Ramachandran plots used for validation of RamaMix. The colors black, red and green correspond to most (large), averaged (medium) and least (narrow) scattered 15 ϕ and ψ values. These synthetic data correspond to five hypothetical residues located in three conformations, the relative weights of conformations being 56.8%, 11.6 % and 31.6 %.

Figure S8

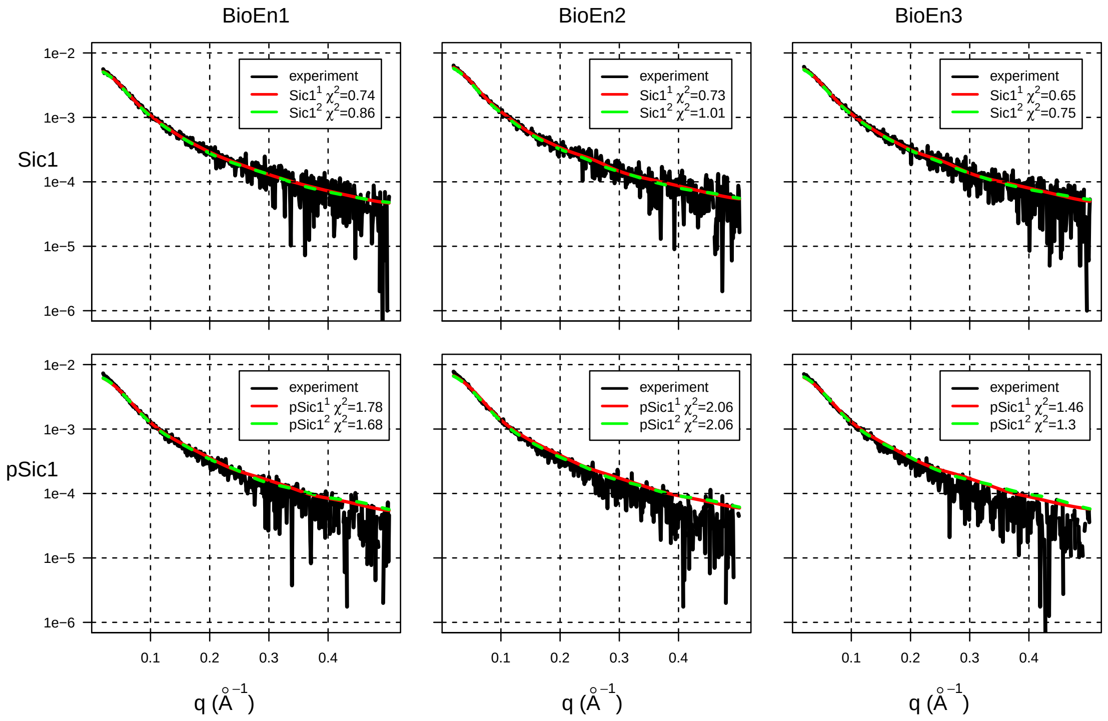

Figure S8: Superimposition of experimental SAXS curves with the reconstructed SAXS curves from the conformations of Sic1 and pSic1 selected by BioEn. The reconstructions of the SAXS curves from the selected conformations are plotted using red and green solid lines, depending on the TAiBP first ($Sic1^1$, $pSic1^1$) or second ($Sic1^2$, $pSic1^2$) run.

Figure S9

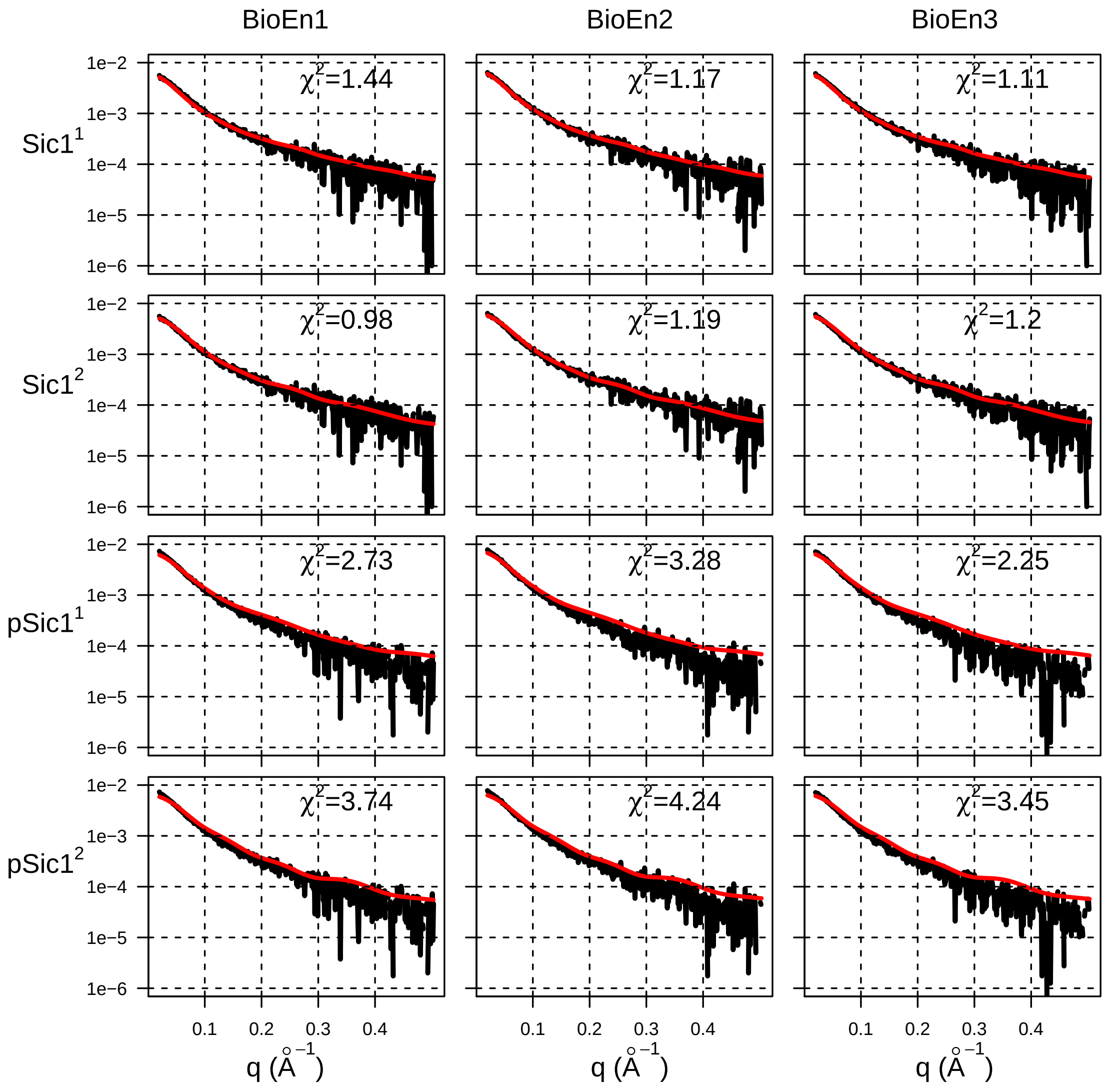

Figure S9: Superimposition of experimental SAXS curves (black) with the reconstructed SAXS curves (red) from the conformations of Sic1 and pSic1 selected by RamaMix.

Figure S10

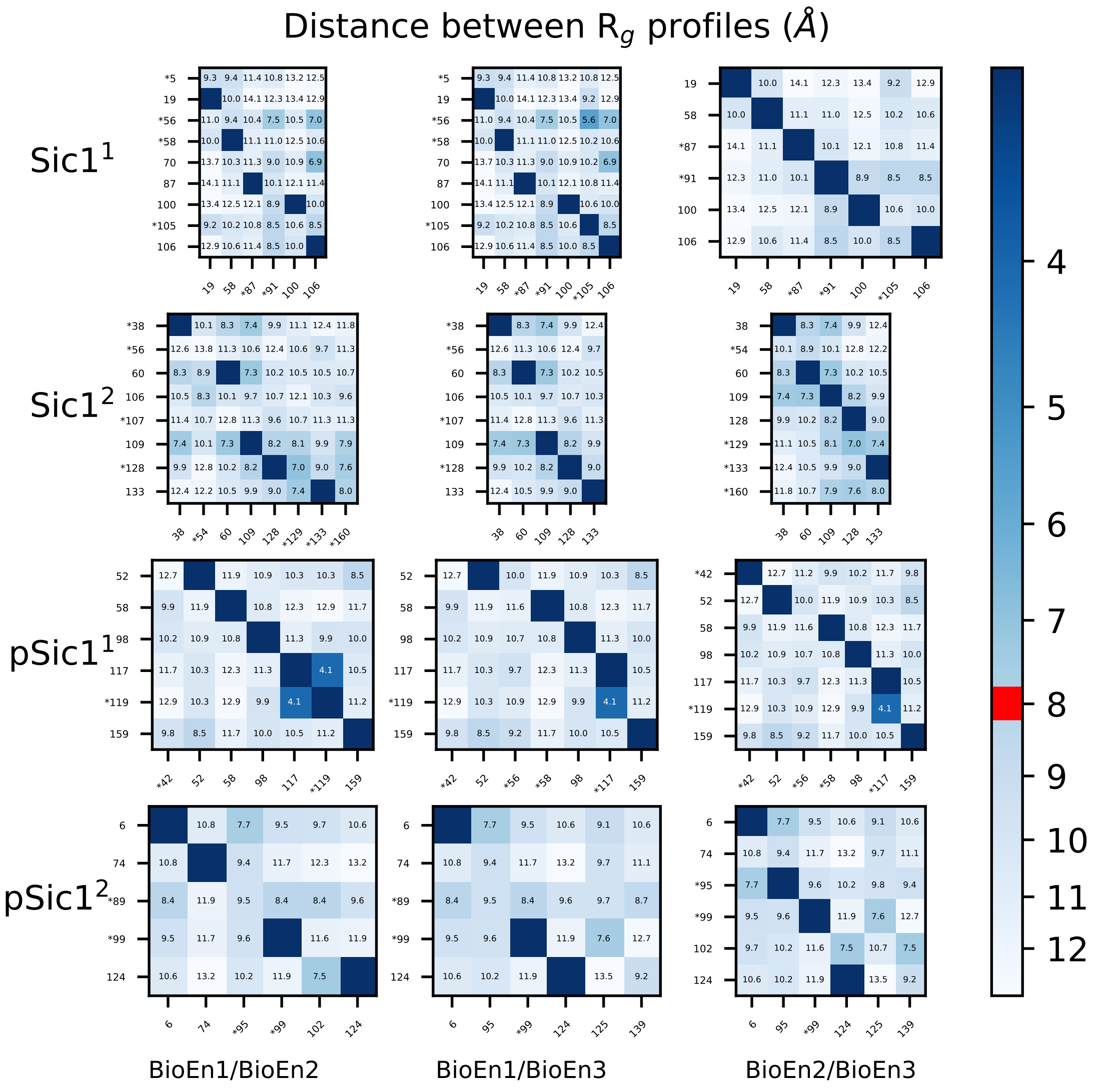

Figure S10: Distances between the profiles P_q (Eq. 11 of the main text) for local gyration radii between the conformations selected from different fittings of SAXS curves (BioEn1,BioEn2, BioEn3). The limit of 8 Å used for the superposed plots of profiles (Figure 5 of the main text) is drawn in red on the scale of distance.

Figure S11

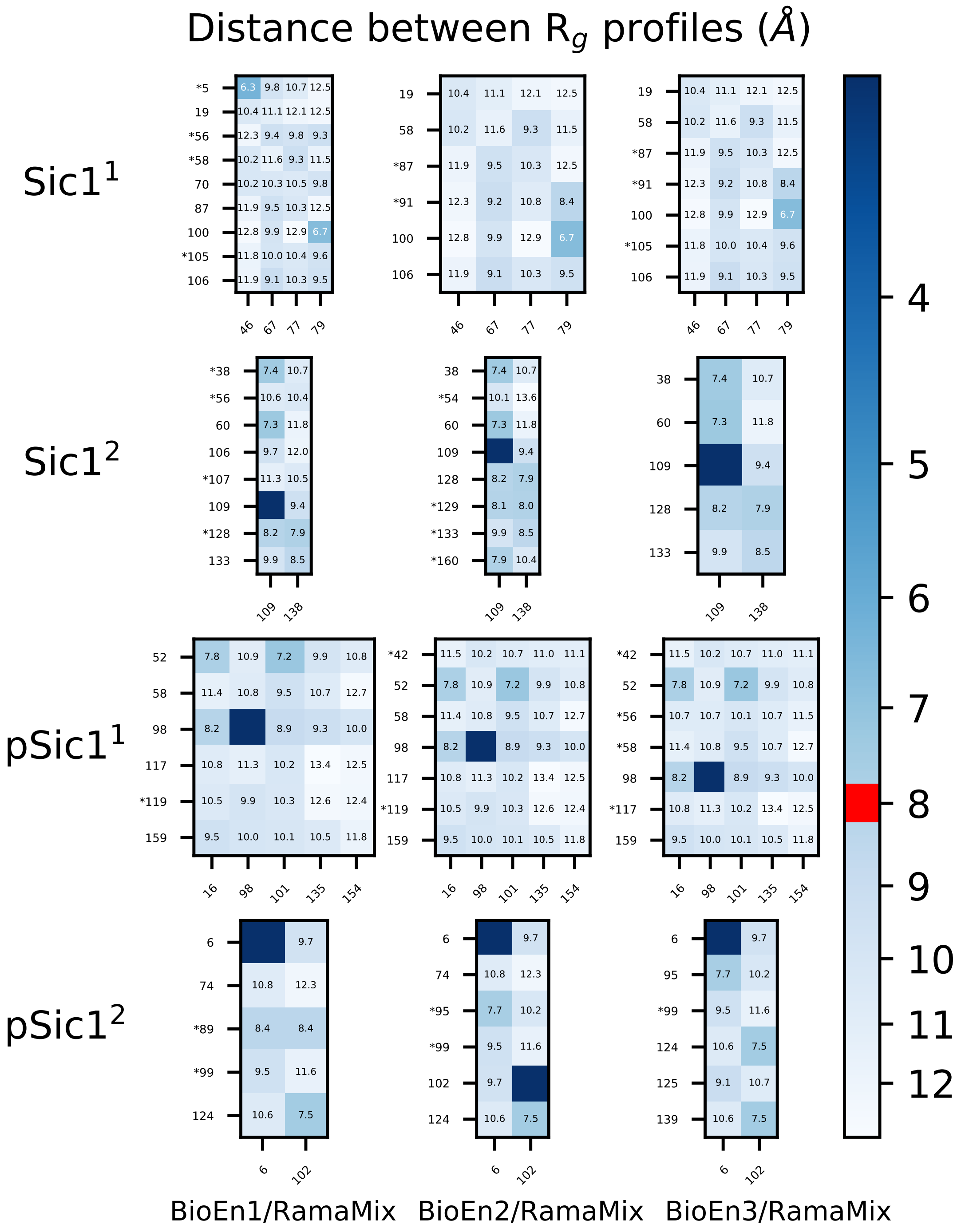

Figure S11: Distances between the profiles P_q (Eq. 11 of the main text) for local gyration radii between the conformations selected from fitting of SAXS curves (BioEn1,BioEn2, BioEn3) and of Ramachandran maps (RamaMix). The limit of 8 Å used for the superposed plots of profiles (Figure 5 of the main text) is drawn in red on the scale of distance.

Figure S12

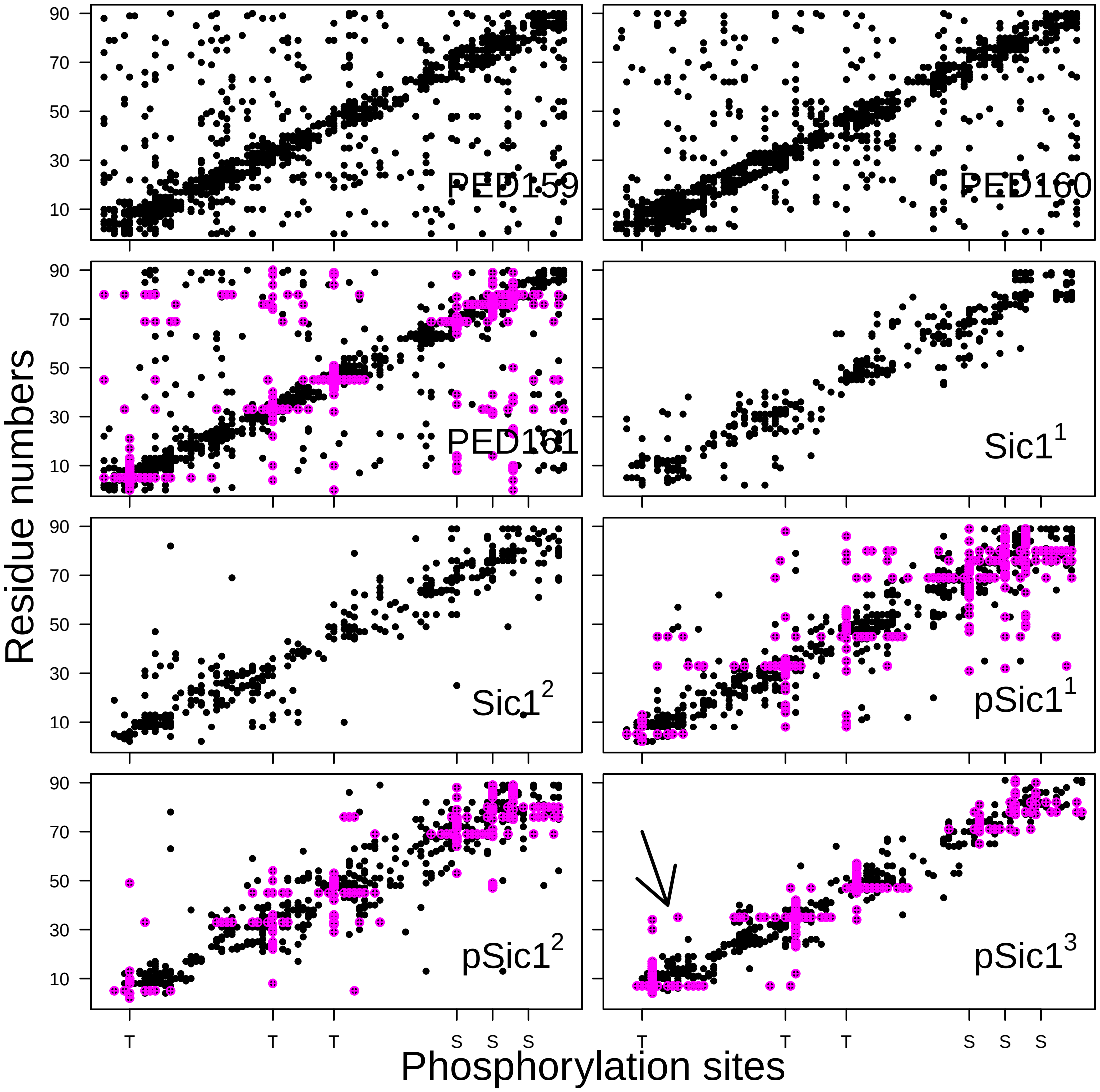

Figure S12: Contact maps displaying cumulative hydrogen bonds observed for the conformation sets PED159(Sic1), PED160(Sic1), PED161(pSic1) refined using MD simulations, as well as for the TAiBP conformational sets $Sic1^1$, $Sic1^2$, $pSic1^1$, $pSic1^2$ and $pSic1^3$. The hydrogen bonds involving sidechains of phosphorylated residues are plotted in magenta, whereas the other hydrogen bonds are plotted in black. An arrow on the contact map of $pSic1^3$ indicates the presence of few long-range hydrogen bonds involving phosphorylated residues.

Fragment	Residue range Sic1[1]	Residue range Sic1[2]	Fragment	Residue range pSic1[1]	Residue range pSic1[2]	Fragment	Residue range pSic1[3]
Pept1	4-12	4-12	Pept1 (pT7)	4-11	4-11	Pept1 (pT7)	4-11
Pept2	10-19	10-19	Pept2	9-15	9-15	Pept2	9-15
Pept3	17-25	17-25	Pept3	13-21	13-21	Pept3	13-21
Pept4	23-32	23-31	Pept4	19-26	19-26	Pept4	19-26
Pept5	30-39	29-38	Pept5	24-32	24-32	Pept5	24-32
Pept6	37-48	36-47	Pept6 (pT35)	30-37	30-37	Pept6 (pT35)	30-37
Pept7	46-56	45-56	Pept7 (pT35)	35-44	35-44	Pept7 (pT35)	35-42
Pept8	54-63	54-63	Pept8 (pT47)	42-51	42-51	Pept8 (pT47)	40-47
Pept9	61-67	61-68	Pept9	49-57	49-58	Pept9	45-51
Pept10	65-71	66-73	Pept10	55-62	56-63	Pept10	49-58
Pept11	69-76	71-79	Pept11	60-66	61-67	Pept11	56-63
Pept12	74-81	77-85	Pept12	64-70	65-71	Pept12	61-67
Pept13	79-87	83-91	Pept13 (pS71)	68-74	69-75	Pept13 (pS71)	65-71
Pept14	85-91	-	Pept14 (pS78)	72-78	73-79	Pept14 (pS78)	69-75
			Pept15 (pS78)	76-82	77-83	Pept15 (pS78)	73-79
			Pept16 (pS82)	80-86	81-87	Pept16 (pS82)	77-83
			Pept17	84-91	85-91	Pept17 (pS82)	81-87
						Pept18	85-91

Table S1: Peptide fragments used for TAiBP runs. The phosphorylated residues in pSic1 are indicated as pS and pT.

ϕ interval	ψ interval
-150 -20	-100 -50
-180 -50	80 180
40 100	0 80

Table S2: Definition of backbone angle generic boxes used for residues of pSic1 on which TALOS-N [1] does not produce a prediction.

Conformations	BioEn1	BioEn2	BioEn3
$Sic1^1$/BioEn1	0.74	3.74	1.45
$Sic1^1$/BioEn2	3.64	0.73	1.5
$Sic1^1$/BioEn3	1.27	1.57	0.65
$Sic1^2$/BioEn1	0.86	4.71	1.98
$Sic1^2$/BioEn2	1.97	1.01	0.76
$Sic1^2$/BioEn3	0.96	2.13	0.75
$pSic1^1$/BioEn1	1.78	4.21	1.59
$pSic1^1$/BioEn2	3.65	2.06	2.53
$pSic1^1$/BioEn3	1.8	3.41	1.46
$pSic1^2$/BioEn1	1.68	4.42	1.51
$pSic1^2$/BioEn2	2.24	2.06	1.59
$pSic1^2$/BioEn3	1.69	3.34	1.3

Table S3: Values of χ^2 between experimental and reconstructed SAXS curves obtained for the various sets of conformations selected by BioEn on Sic1 and pSic1. The Table columns are labeled with experimental SAXS curves, and the Table rows are labeled with the sets of conformations selected from the fitting of SAXS curves.

Residue position	order
first	N, H1, H2, CA, N, HA, CA, C
inner	N, -O, -CA, -C, N, CA, C, +N,<br>-C, N, CA, H1, N, CA, C, HA, C, CA
last	N, -O, -CA, -C, N, CA, C,<br>-C, N, CA, H1, N, CA, C, HA,<br>C, CA, O, C, O2

Table S4: Atom re-ordering used during the iBP calculation step within the first, the last and the inner residues of the peptide fragment. The order is described by the list of atoms names, the signs ”-” and ”+” describing atoms located in the previous and the next residues in the primary sequence.

A. pSic1^3	conformation numbers	populations percentages	conformation numbers	populations percentages	conformation numbers	populations percentages
	**270**	51.6 ± 1.3e-3	**270**	42.9 ± 1.1e-3	**270**	40.2 ± 0.3
	**240**	39.6 ± 1.2e-3	**240**	45.7 ± 3.6e-4	**240**	43.6 ± 7.2e-2
	**249**	8.8 ± 3.9e-4	**249**	11.4 ± 2.0e-4	**249**	15.2 ± 6.1e-2
Average final $\chi 2$		0.9		1.2		0.7
Average final S_{KL}		-2.1e-9		-3.4e-8		-2.9e-10
B. pSic1^{13}	conformation numbers	populations percentages	conformation numbers	populations percentages	conformation numbers	populations percentages
	**239**	47.2 ± 2.3e-3	**239**	44.1 ± 8.6e-4	**239**	50.5 ± 1.8e-3
	**249**	8.6 ± 6.4e-4	**249**	12.0 ± 4.2e-4	**249**	14.1 ± 1.9e-4
	**52**	29.0 ± 5.4e-4	**52**	26.3 ± 9.3e-4	**52**	19.4 ± 4.8e-4
	**54**	15.2 ± 1.1e-3	**54**	17.6 ± 7.2e-4	**54**	15.9 ± 7.4e-4
Average final $\chi 2$		0.8		0.9		0.7
Average final S_{KL}		-1.5e-9		-5.4e-9		-2.6e-8
C. pSic1^{23}	conformation numbers	populations percentages	conformation numbers	populations percentages	conformation numbers	populations percentages
	**239**	31.6 ± 2.1e-3	**239**	25.7 ± 9.0	**239**	39.6 ± 6.1e-4
	**249**	28.5 ± 8.5e-4	**249**	30.3 ± 5.9	**249**	31.2 ± 3.3e-4
			240	2.1 ± 6.3		
			316	3.1 ± 9.1		
			47	1.6 ± 4.9		
	**74**	16.0 ± 5.4e-4	**74**	16.5 ± 5.5	**74**	16.6 ± 2.3e-4
	**99**	23.9 ± 5.4e-4	**99**	20.7 ± 3.2	**99**	12.6 ± 6.9e-4
Average final $\chi 2$		0.8		0.9		0.7
Average final S_{KL}		-3.2e-9		-4.6e-9		-2.9e-10

Table S5: Conformations and populations selected using BioEn 0.1.1 [31] on the three sets of SAXS curves. The conformations were generated by the runs pSic1^3 and also pooled with pSic1^1 and pSic1^2 to produce pSic1^{13} pSic1^{23}. For each SAXS curve and set of protein conformations, after ten runs starting from random values of populations and performed on the whole set of conformations, all conformations for which the sum of populations over the ten runs was larger than 0.01 were gathered, and a second run of ten additional BioEn calculations was performed on this reduced set of conformations. The average and standard deviation values of populations obtained for each selected conformation from the second set of BioEn runs, are given in the Table, along with the final average values of reduced χ^2 and of entropy S_{KL}. The labels of conformations selected in at least two runs are written in bold. Labels larger than 200 correspond to conformations from pSic1^3.

A. pSic1^{3}	conformation numbers	populations percentages
	**240**	28.5 ± 3.2e-5
	247	39.5 ± 3.8e-5
	**249**	32.0 ± 3.1e-5
B. pSic1^{13}	conformation numbers	populations percentages
	247	39.5 ± 2.7
	**249**	31.7 ± 3.0
	240	28.8 ± 0.9
C. pSic1^{23}	conformation numbers	populations percentages
	240	28.8 ± 1.0
	247	39.4 ± 2.6
	**249**	31.7 ± 2.8

Table S6: Conformations and populations selected by fitting of the Ramachandran maps using RamaMix. For each set of protein conformations, 100 runs were performed starting from random values for the populations. The few optimizations which did not converge, were discarded: 2 for pSic1^{13} and for pSic1^{23}. The backbone angles ϕ and ψ were allowed to move up to 15°. The populations of conformations for the converged runs were averaged and these mean values are given as percentages in the Table along with the corresponding standard deviation values. The labels of conformations also selected by BioEn are written in bold. Labels larger than 200 correspond to conformations from pSic1^{3}.

References

[1] Y. Shen and A. Bax. Protein structural information derived from NMR chemical shift with the neural network program TALOS-N. *Methods Mol Biol*, 1260:17–32, 2015.

[2] L. Liberti, C. Lavor, and A. Mucherino. The discretizable molecular distance geometry problem seems easier on proteins. *Distance Geometry: Theory, Methods and Applications. Mucherino, Lavor, Liberti, Maculan (eds.)*, pages 47–60, 2014.

[3] C. Lavor, R. Alves, W. Figueiredo, A. Petraglia, and N. Maculan. Clifford Algebra and the Discretizable Molecular Distance Geometry Problem. *Adv. Appl. Clifford Algebras*, 25:925–942, 2015.

[4] B. Worley, F. Delhommel, F. Cordier, T. Malliavin, B. Bardiaux, N. Wolff, M. Nilges, C. Lavor, and L. Liberti. Tuning interval Branch-and-Prune for protein structure determination. *Journal of Global Optimization*, 72:109–127, 2018.

[5] R. Engh and R. Huber. Accurate bond and angle parameters for X-ray protein structure refinement. *Acta Crystallogr A*, 47:392–400, 1991.

[6] T. E. Malliavin. Tandem domain structure determination based on a systematic enumeration of conformations. *Sci Rep*, 11:16925, 2021.

[7] W. Humphrey, A. Dalke, and K. Schulten. VMD – Visual Molecular Dynamics. *Journal of Molecular Graphics*, 14:33–38, 1996.

[8] T. Kohonen. Self-organized formation of topologically correct feature maps. *Biol Cybern*, 43:59–69, 1982.

[9] T. Kohonen. Self-organizing maps. *Springer Series in Information Sciences, Heidelberg, Germany.*, 2001.

[10] L. Miri, G. Bouvier, A. Kettani, A. Mikou, L. Wakrim, M. Nilges, and T. E. Malliavin. Stabilization of the integrase-DNA complex by Mg^{2+} ions and prediction of key residues for binding HIV-1 integrase inhibitors. *Proteins*, 82(3):466–478, Mar 2014.

[11] G. Bouvier, N. Duclert-Savatier, N. Desdouits, D. Meziane-Cherif, A. Blondel, P. Courvalin, M. Nilges, and T. Malliavin. Functional motions modulating VanA ligand binding unraveled by self-organizing maps. *J Chem Inf Model*, 54:289–301, 2014.

[12] Y. Spill, G. Bouvier, and M. Nilges. A convective replica-exchange method for sampling new energy basins. *J Comput Chem*, 34:132–140, 2013.

[13] G. W. Gomes, M. Krzeminski, A. Namini, E. W. Martin, T. Mittag, T. Head-Gordon, J. D. Forman-Kay, and C. C. Gradinaru. Conformational Ensembles of an Intrinsically Disordered Protein Consistent with NMR, SAXS, and Single-Molecule FRET. *J Am Chem Soc*, 142:15697–15710, 2020.

[14] J. Phillips, R. Braun, W. Wang, J. Gumbart, E. Tajkhorshid, E. Villa, C. Chipot, R. Skeel, L. Kale, and K. Schulten. Scalable molecular dynamics with NAMD. *J Comput Chem*, 26:1781–1802, 2005.

[15] R. B. Best, X. Zhu, J. Shim, P. E. Lopes, J. Mittal, M. Feig, and A. D. Mackerell. Optimization of the additive CHARMM all-atom protein force field targeting improved sampling of the backbone ϕ and ψ and side-chain $\chi(1)$ and $\chi(2)$ dihedral angles. *J Chem Theory Comput*, 8:3257–3273, 2012.

[16] J. Huang, S. Rauscher, G. Nawrocki, T. Ran, M. Feig, B. L. de Groot, H. Grubmüller, and A. D. MacKerell. CHARMM36m: an improved force field for folded and intrinsically disordered proteins. *Nat Methods*, 14:71–73, 2017.

[17] D. E. Tanner, K. Y. Chan, J. C. Phillips, and K. Schulten. Parallel Generalized Born Implicit Solvent Calculations with NAMD. *J Chem Theory Comput*, 7(11):3635–3642, Nov 2011.

[18] J. Ryckaert, G. Ciccotti, and H. Berendsen. Numerical integration of the cartesian equations of motion of a system with constraints and Molecular dynamics of n-alkanes. *J. Comput. Phys.*, 23:327–341, 1977.

[19] H. Andersen. Rattle: a "Velocity" Version of the Shake Algorithm for Molecular Dynamics Calculations. *J Comp Phys*, 52:24–34, 1983.

[20] D. Frenkel and B. Smit. *Understanding molecular simulation: from algorithms to applications.* Academic press, San Diego, California, 2002.

[21] E. L. Lehmann and G. Casella. *Theory of point estimation.* Springer Texts in Statistics. Springer-Verlag, New York, NY, 2nd edition, 1998.

[22] G. J. McLachlan and T. Krishnan. *The EM Algorithm and Extensions.* Wiley series in probability and statistics. John Wiley and Sons, Inc., 1997.

[23] C. M. Bishop. *Pattern Recognition and Machine Learning (Information Science and Statistics).* Springer-Verlag, Berlin, Heidelberg, 2006.

[24] K. V. Mardia, G. Hughes, C. C. Taylor, and H. Singh. A multivariate von Mises distribution with applications to bioinformatics. *Canadian Journal of Statistics*, 36(1):99–109, 2008.

[25] H. Singh, V. Hnizdo, and E. Demchuk. Probabilistic model for two dependent circular variables. *Biometrika*, 89(3):719–723, 2002.

[26] K. V. Mardia, C. C. Taylor, and G. K. Subramaniam. Protein Bioinformatics and Mixtures of Bivariate von Mises Distributions for Angular Data. *Biometrics*, 63(2):505–512, 2007.

[27] W. Boomsma, K. V. Mardia, C. C. Taylor, J. Ferkinghoff-Borg, A. Krogh, and T. Hamelryck. A generative, probabilistic model of local protein structure. *Proc Natl Acad Sci U S A*, 105:8932–8937, 2008.

[28] R. H. Byrd, P. Lu, J. Nocedal, and C. Zhu. A limited memory algorithm for bound constrained optimization. *SIAM Journal on Scientific Computing*, 16:1190–1208, September 1995.

[29] O. Cappé, E. Moulines, and T. Rydén. *Inference in hidden Markov models.* Springer Verlag, New York, NY, 2005.

[30] D. E. Amos. Computation of modified Bessel functions and their ratios. *Mathematics of Computation*, 28(125):239–251, 1974.

[31] J. Köfinger, L. S. Stelzl, K. Reuter, C. Allande, K. Reichel, and G. Hummer. Efficient Ensemble Refinement by Reweighting. *J Chem Theory Comput*, 15(5):3390–3401, May 2019.
